# Inhibiting the activation of MAPK (ERK1/2) in stressed Müller cells prevents photoreceptor degeneration

**DOI:** 10.7150/thno.71038

**Published:** 2022-09-11

**Authors:** Shaoxue Zeng, Ting Zhang, Yingying Chen, Joshua Chu-Tan, Kaiyu Jin, So-Ra Lee, Michelle X Yam, Michele C Madigan, Nilisha Fernando, Adrian Cioanca, Fanfan Zhou, Meidong Zhu, Junjun Zhang, Riccardo Natoli, Xiaohui Fan, Ling Zhu, Mark C Gillies

**Affiliations:** 1 Save Sight Institute, Faculty of Medicine and Health, The University of Sydney, Sydney, NSW 2006, Australia.; 2 Department of Ophthalmology, West China Hospital, Sichuan University, Chengdu, Sichuan 610041, China.; 3 The John Curtin School of Medical Research, The Australian National University, Canberra, ACT 2601, Australia.; 4 The Australian National University Medical School, Canberra, ACT 2601, Australia.; 5 Pharmaceutical Informatics Institute, College of Pharmaceutical Sciences, Zhejiang University, Hangzhou, Zhejiang 310058, China.; 6 School of Optometry and Vision Sciences, University of New South Wales, Sydney, NSW 2052, Australia.; 7 Neuro-Immune Regulome Unit, National Eye Institute, National Institutes of Health, Bethesda, MD, 20892, United States.; 8 School of Pharmacy, The University of Sydney, Sydney, NSW 2006, Australia.; 9 New South Wales Organ and Tissue Donation Service, New South Wales Tissue Bank, Sydney Eye Hospital, Sydney, NSW 2006, Australia.

**Keywords:** PD98059, pERK1/2 inhibition, Müller cells, photoreceptor degeneration

## Abstract

**Rationale:** Müller cells play an essential role in maintaining the health of retinal photoreceptors. Dysfunction of stressed Müller cells often results in photoreceptor degeneration. However, how these cells communicate under stress and the signalling pathways involved remain unclear. In this study, we inhibited the MAPK (ERK1/2) signalling, mainly activated in Müller cells, evaluated the protective effects on the photoreceptors and further explored the signalling communication between stressed Müller cells and degenerating photoreceptors.

**Methods:** We evaluated the changes of MAPK (ERK1/2) signalling and its downstream targets in human retinal explants treated with PD98059, a specific phosphorylated ERK1/2 inhibitor, by western blot and immunostaining. We further assessed photoreceptor degeneration by TUNEL staining and outer nuclear layer thickness. We also injected PD98059 into the eyes of mice exposed to photo-oxidative stress. We evaluated the protective effects on photoreceptor degeneration by optical coherence tomography (OCT) and electroretinography (ERG). The crosstalk between Müller cells and photoreceptors was further dissected based on the changes of transcription factors by RNA sequencing and protein profiles of multiple signalling pathways.

**Results:** We found that MAPK (ERK1/2) signalling was mainly activated in Müller cells under stress, both *ex vivo* and* in vivo*. PD98059 inhibited the phosphorylation of ERK1/2, reduced expression of the gliotic marker glial fibrillary acidic protein (GFAP) in Müller cells and increased levels of the neuroprotective factor, interphotoreceptor retinoid-binding protein (IRBP) in photoreceptors. Inhibition of pERK1/2 also reduced retinal photo-oxidative damage in mice retinas assessed by OCT and ERG. We also identified that the JAK/STAT3 signalling pathway might mediate signalling transduction from Müller cells to photoreceptors.

**Conclusion:** MAPK (ERK1/2) deactivation through chemical inhibition, mainly in stressed Müller cells, can alleviate gliosis in Müller cells and restore the expression of IRBP in photoreceptors, which appears to prevent retinal degeneration. Our findings suggested a new way to prevent photoreceptor degeneration by manipulating the stress response in Müller cells.

## Introduction

Müller cells play an essential role in maintaining the health of photoreceptors 1. Extending the thickness of the retina from the inner to the outer limiting membrane (OLM), they form columnar units with other retinal cells allowing for anatomical and functional cellular interactions within the retina 2. Müller cells interact anatomically with photoreceptors at the OLM comprised of the junctional complexes 3. Müller cells regulate ion and water homeostasis within the whole retina 4-7. They also share a “metabolic symbiosis” with neurons by exchanging glycolytic metabolites and carbon dioxide 1, 8. Müller cells maintain retinal redox balance by generating antioxidants such as glutathione or regulating antioxidant-related genes such as nuclear factor erythroid 2-related factor 2, peroxiredoxin 6, nicotinamide adenine dinucleotide phosphatase-quinone oxidoreductase, superoxide dismutase2 and glutathione peroxidase 1 9-11. They secrete neurotrophic factors like brain-derived neurotrophic factor, ciliary neurotrophic factor (CNTF), nerve growth factor, neurotrophin-3 and neurotrophin-4 12-14. Finally, Müller cells can scavenge and recycle neurotransmitters such as glutamate, γ-Aminobutyric acid and phagocyte photoreceptor debris, thus preventing potential neurotoxicity 15-17.

Müller cell dysfunction is commonly associated with photoreceptor degeneration and has been reported in both *in vitro* and *in vivo* models of diabetic retinopathy 18, retinitis pigmentosa 19, Leber hereditary optic neuropathy 20 and human Macular Telangiectasia type 2 21, 22. We have also previously reported photoreceptor cell degeneration in a murine model with induced Müller cell disruption 23.

Müller cells produce cytokines such as interleukin-1 beta (IL-1 beta), leukemia inhibitory factor (LIF) and interleukin-6 (IL-6) in response to stress 24-27. These cytokines can directly activate STAT3 signalling, which may signal other retinal cell types, including photoreceptors 28-32. While signalling pathways such as p38 mitogen-activated protein kinase (MAPK) have been implicated in the interaction between Müller cells and photoreceptors 33, precisely how Müller cells regulate and sustain photoreceptors remains unclear.

MAPK has three major subfamilies in mammalian cells: extracellular signal-regulated kinase 1/2 (ERK1/2), c-Jun NH2-terminal kinase and p38 MAPK 34. It is widely involved in cell proliferation, differentiation and apoptosis 35. Diverse extracellular stimuli, including growth factors, mitogens, hormones, cytokines and cellular stressors can activate MAPK signalling 36, 37. PD98059 inhibits the phosphorylation of ERK1/2 by binding to inactive forms of MAPK/ERK kinase, thus preventing subsequent phosphorylation 38, 39. It has been reported to protect the brain through antioxidant and anti-apoptotic functions 40. The present study used PD98059 to inhibit the activation of MAPK (ERK1/2) signalling and explored the molecular changes in photoreceptors in response to this. We found MAPK (ERK1/2) signalling inhibition protected photoreceptors from degeneration and restored part of the electrical response of the photoreceptors. These studies further elucidate the potential interactions between Müller cells and photoreceptors under stress and identify new treatment targets for retinal diseases.

## Results

### MAPK (ERK1/2) signalling is activated in human Müller cells under stress

**Figure 1:**
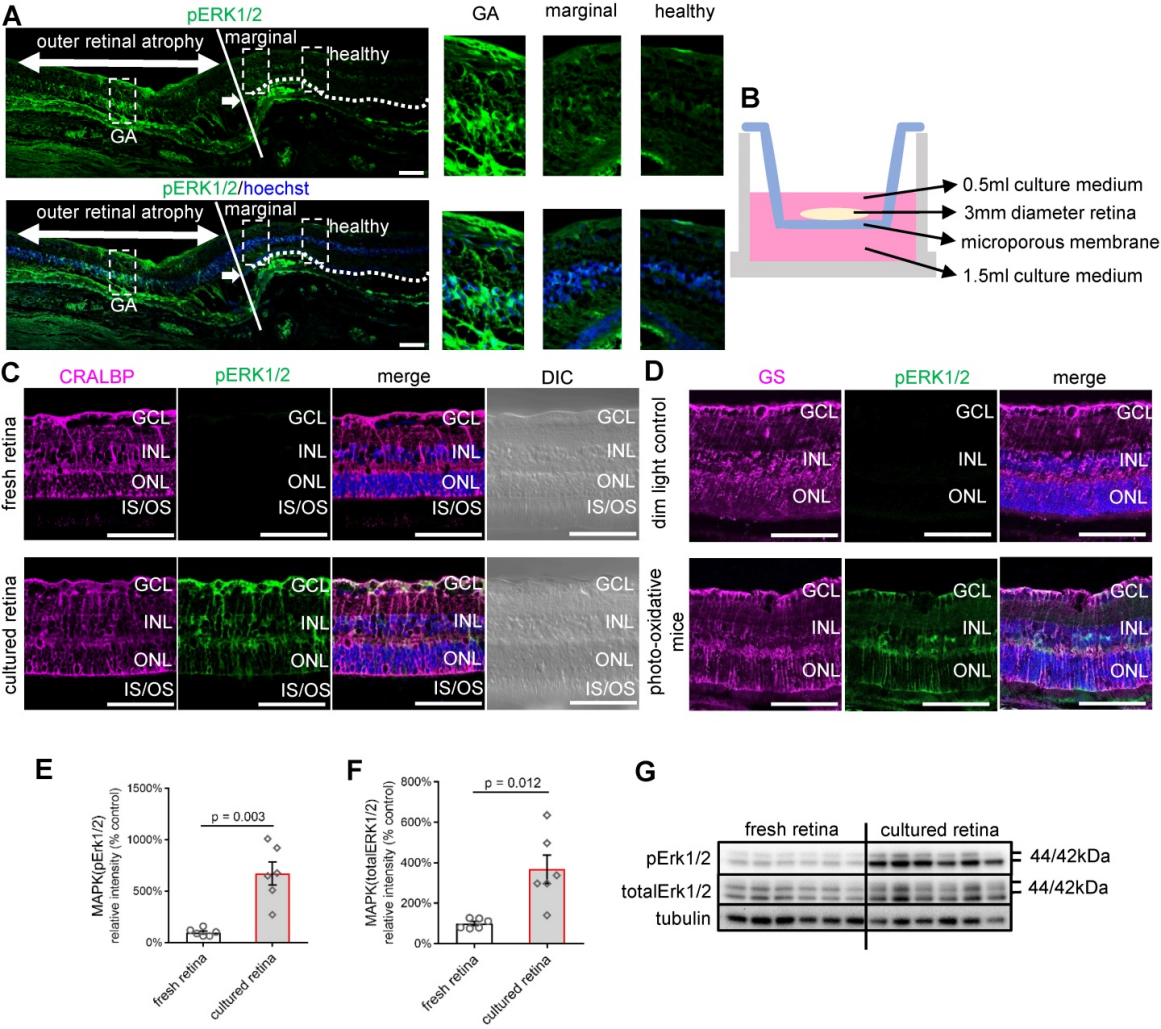
** MAPK (pERK1/2) activation in human retinal disease, human retinal explants and retinas of photo-oxidative stressed mice. (A)** Paraffin sections of human retina from an eye with GA immunostained for pERK1/2 (green) and Hoechst (blue, cell nuclei staining). The white arrow indicates the transition point where the ONL disappears related to the loss and degeneration of the photoreceptors to the left of the image and RPE. The white line indicates the edge of the atrophic lesion in the middle. The dotted white line on the right indicates the OLM extending to the right in an area of the retina without atrophy. High power images of the GA area, transition area, and intact retina are also shown. **(B)** Schematic diagram of a retinal explant growing on the insert membrane of a transwell (12 well plate). **(C)** Vibratome cross-sections of the freshly fixed human retina or cultured human retinal explants stained for CRALBP (magenta), pERK1/2 (green) and Hoechst (blue, cell nuclei staining). **(D)** Cryosections of mice eyes stained for GS (magenta), pERK1/2 (green) and Hoechst (blue, cell nuclei staining) in 4-day photo-oxidative stressed model or dim light control. **(E)** Summary results of western blot of pERK1/2 in the fresh retina or human retinal explants after 24 h culture (pERK1/2 is normalised by total ERK1/2). Data are shown as the mean ± SEM. n = 6. **(F)** Summary results of western blot of total ERK1/2 in the fresh retina or human retinal explants after 24 h culture (total ERK1/2 is normalised by Tubulin). Data are shown as the mean ± SEM. n = 6. **(G)** Western blot image of pERK1/2, total ERK1/2 and Tubulin in the fresh retina or human retinal explants after 24 h culture. DIC: Differential Interference Contrast. GCL: ganglion cell layer. INL: inner nuclear layer. ONL: outer nuclear layer. OLM: outer limiting membrane. IS: inner segments. OS: outer segments. RPE: retinal pigment epithelium. CRALBP: cellular retinaldehyde-binding protein. GS: Glutamine Synthetase. GA: geographic atrophy. ERK1/2: extracellular signal-regulated kinase1/2. pEKR1/2: phosphorylated ERK1/2. Scale Bar = 100 µm.

The activation of MAPK (ERK1/2) signalling, predominantly in Müller cells, is an established response to stress 41-45. We first explored MAPK (ERK1/2) activation in a human retina with geographic atrophy (GA) by immunostaining (Figure 1A). Fundus image of the post-mortem donor eye showed GA in the macula region (Figure S1). The phosphorylated ERK1/2 (pERK1/2) antibody we used identified phosphorylated forms of ERK1 and ERK2, markers for MAPK (ERK1/2) activation. In the section, an area of outer retinal atrophy is seen on the left with the loss of retinal photoreceptors, outer nuclear layer (ONL) and retinal pigment epithelium (RPE) (Figure 1A). The edge of the atrophic lesion is indicated by the white line in the middle. The dotted white line on the right indicates the OLM extending in the retina to the right, which shows all layers and no atrophy. Interestingly, we found patches of phosphorylation of ERK1/2 along with the disappearance of the ONL. The ERK1/2 was strongly phosphorylated in the degenerative area, gradually reduced in the marginal area, and extremely weak in the healthy area (Figure 1A). Representative high power images of the relatively healthy retina on the right, the area of outer retinal degeneration on the left and the transition between are presented (Figure 1A). This is also indicated on the merged and differential interference contrast (DIC) images (Figure S2). These observations suggest that activation of pERK1/2 is closely related to photoreceptor degeneration in humans.

We then studied whether pERK1/2 was activated in human retinal explants, which may be considered continuously under stress when cultured *ex vivo*. Human retinal explants from the mid-peripheral retina (3 mm diameter from the mid-point between the edge of the *macula lutea* and the *ora serrata*) of the same donor were either cultured in Neurobasal-A medium for 24 h (Figure 1B) 46 or processed freshly for vibratome cross-sections. The staining of pERK1/2 was significantly stronger in the cultured retina than in freshly isolated retinal punches (Figure 1C). pERK1/2 staining colocalised with the Müller cell marker cellular retinaldehyde-binding protein (CRALBP) (Figure 1C). pERK1/2 was stained throughout the entire Müller cell body, from the OLM to the inner limiting membranes (ILM), including cell processes in the inner plexiform layer (IPL), somas in the inner nuclear layer (INL) and apical processes in the outer plexiform layer. Western blot confirmed the activation of pERK1/2 in *ex vivo* culture compared with fresh retina (Figure 1E-G). We also found increased staining of pERK1/2 in retinal cryosections of mice subjected to photo-oxidative stress, which is an established model with both Müller cell gliosis and photoreceptor degeneration 47 (Figure 1D). The staining of pERK1/2 was also colocalised with Müller cell marker Glutamine Synthetase (GS) in the mice model. These findings suggest retinal stress can activate pERK1/2, mainly in Müller cells.

### PD98059 inhibited phosphorylation of ERK1/2 in Müller cells

**Figure 2:**
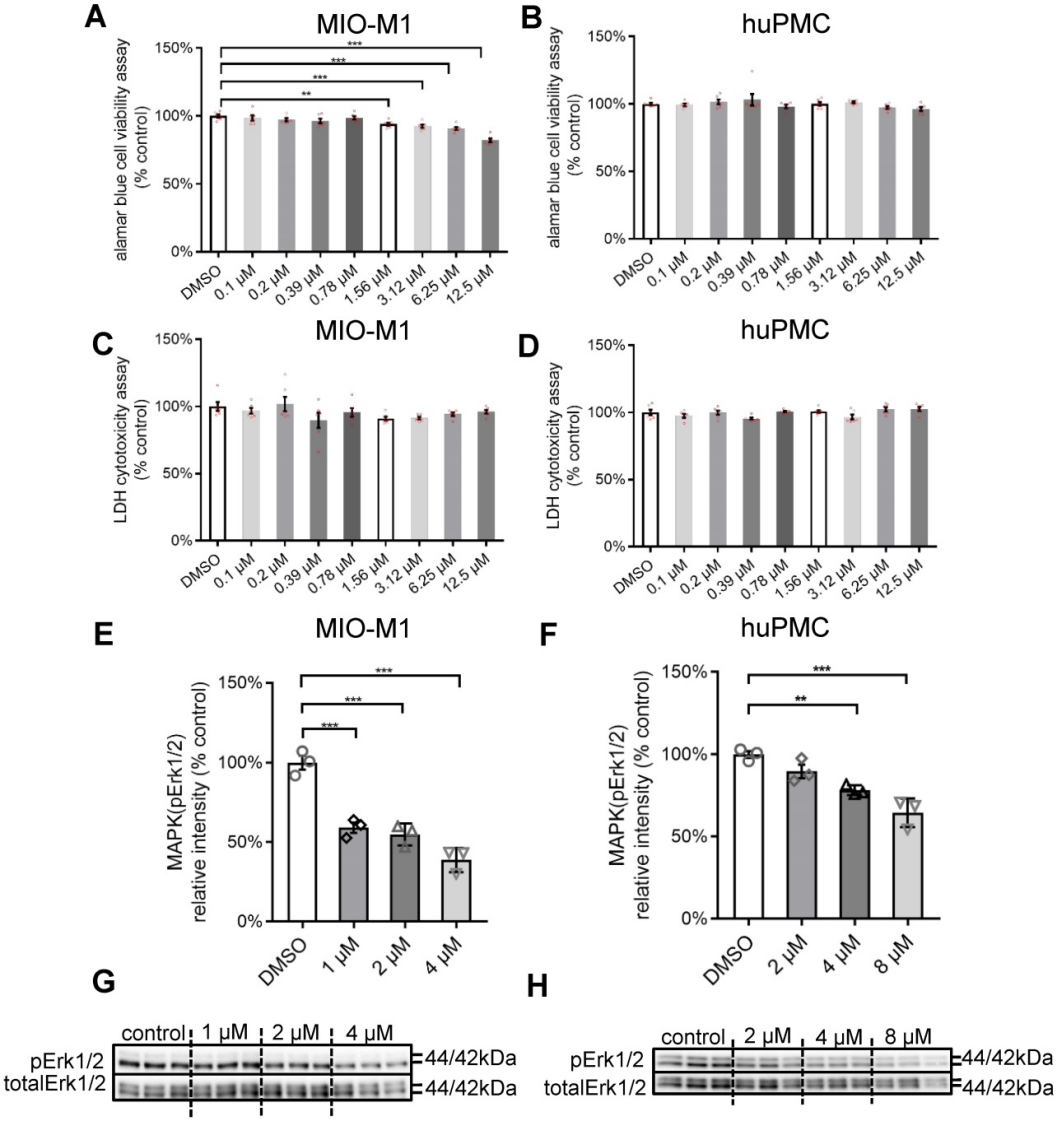
** Safety concentration and inhibition efficiency of PD98059 (pERK1/2 inhibitor) *in vitro.* (A)** Alamar Blue Assay of cell viability in MIO-M1 cells after 22 h treatment + 4h Alamar Blue Assay incubation with different concentrations of PD98059. Data are shown as the mean ± SEM. n = 6. **(B)** Alamar Blue Assay of cell viability in huPMC cells after 22 h treatment + 4h Alamar Blue Assay incubation with different concentrations of PD98059. Data are shown as the mean ± SEM. n = 6. **(C)** LDH cell cytotoxicity assay in MIO-M1 cells after 22 h treatment with different concentrations of PD98059. Data are shown as the mean ± SEM. n = 6. **(D)** LDH cell cytotoxicity assay in huPMC cells after 22 h treatment with different concentrations of PD98059. Data are shown as the mean ± SEM. n = 6. Data are shown as the mean ± SEM. n = 3. **(E)** Summary results of western blot of pERK1/2 and total ERK1/2 in MIO-M1 under 4 h treatment of PD98059 with different concentrations. Data are shown as the mean ± SEM. n = 3. **(F)** Summary results of western blot of pERK1/2 and total ERK1/2 in huPMCs under 4 h treatment of PD98059 with different concentrations. Data are shown as the mean ± SEM. n = 3. **(G)** Western blot image of pERK1/2 and total ERK1/2 in MIO under 4 h treatment of PD98059 with different concentrations. n = 3. **(H)** Western blot image of pERK1/2 and total ERK1/2 in huPMCs under 4 h treatment of PD98059 with different concentrations. n = 3 (**: p < 0.01; ***: p < 0.001). ERK1/2: extracellular signal-regulated kinase1/2. pEKR1/2: phosphorylated ERK1/2. MIO-M1: Moorfields/Institute of Ophthalmology-Müller 1. huPMCs: human primary Müller cells. DMSO: dimethyl sulfoxide.

The activation of MAPK signalling may have pro- or anti-apoptotic mechanisms 48. We studied the effects of PD98059, a specific pERK1/2 inhibitor, in Moorfields/Institute of Ophthalmology-Müller 1 (MIO-M1) cells, human primary Müller cells (huPMCs) and Y79 photoreceptor-derived retinoblastoma cells. We tested the safety (Alamar Blue Cell Viability Assay for metabolic activity, Lactate Dehydrogenase (LDH) assay for cell death) and inhibition efficiency of PD98059 (0.1 µM, 0.2 µM, 0.39 µM, 0.78 µM, 1.56 µM, 3.12 µM, 6.25 µM and 12.5 µM) for 22 h. PD98059 significantly inhibited cellular metabolic activity at a concentration of 1.56 µM or higher for MIO-M1 cells (Figure 2A) and 3.12 µM or higher for Y79 cells (Figure S3A). We found no significant inhibition of cell metabolic activity in huPMCs with up to 12.5 µM PD98059 treatment (Figure 2B). We found no significant cellular cytotoxicity in MIO-M1 cells (Figure 2C), Y79 cells (Figure S3B) and huPMCs (Figure 2D) cells cultured at any concentrations of PD98059 up to 12.5 µM. These data suggest that 12.5 µM PD98059 was within the safe concentration for Müller cells and photoreceptors.

We further studied the inhibition efficiency of various concentrations of PD98059 in MIO-M1 cells and huPMCs by western blot (Figure 2E-H). PD98059 significantly inhibited the level of pERK1/2 in MIO-M1 cells (Figure 2E, G) and huPMCs (Figure 2F, H) dose-dependently, indicating inhibition of MAPK (ERK1/2) signalling in Müller cells.

### PD98059 inhibited phosphorylation of ERK1/2, predominantly in Müller cells, in human retinal explant

**Figure 3:**
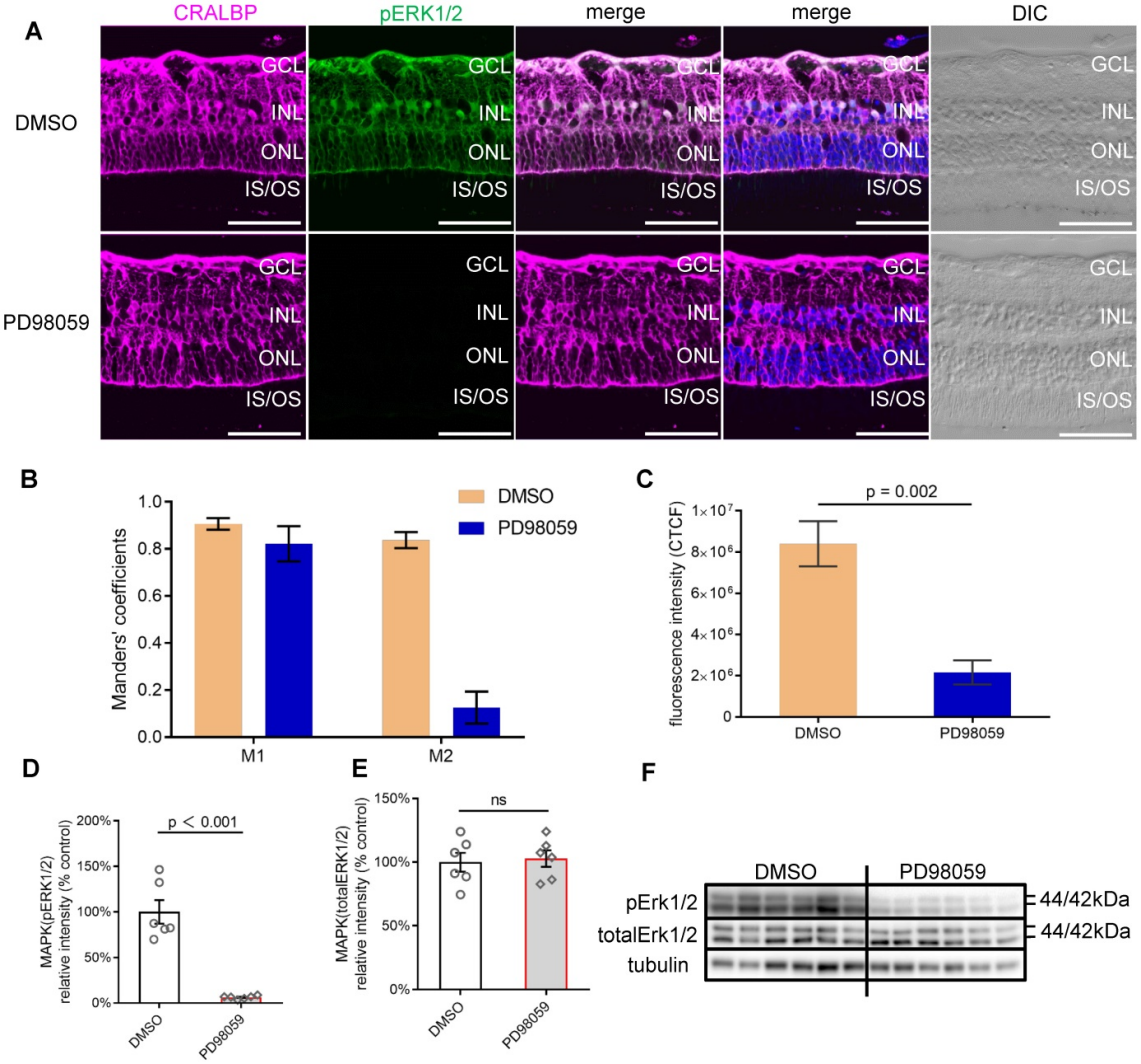
** PD98059 inhibited MAPK (pERK1/2) activation in human retinal explant. (A)** Vibratome cross-sections of human retinal explants stained for CRALBP (magenta), pERK1/2 (green) and Hoechst (blue, cell nuclei staining) in DMSO and PD98059 group. **(B)** The colocalisation of pERK1/2 and CRALBP DMSO and PD98059 treated human retinal explants by Manders' correlation coefficients M1 and M2 (M1 indicates the overlap of pERK1/2 signal with CRALBP signal. M2 indicates the overlap of CRALBP signal with pERK1/2 signal), n = 5. **(C)** Quantification of pERK1/2 fluorescence staining intensity presented as CTCF. n = 5. **(D)** Summary results of western blot of pERK1/2 in DMSO and PD98059 treated human retinal explants (pERK1/2 is normalised by total ERK1/2). Data are shown as the mean ± SEM. n = 6. **(E)** Summary results of western blot of total ERK1/2 in in control or MAPK inhibition human retinal explants (total ERK1/2 is normalised by Tubulin). Data are shown as the mean ± SEM. n = 6. **(F)** Western blot image of pERK1/2, total ERK1/2 and Tubulin in control or MAPK inhibition human retinal explants. DIC: Differential Interference Contrast. GCL: ganglion cell layer. INL: inner nuclear layer. ONL: outer nuclear layer. IS: inner segments. OS: outer segments. CRALBP: cellular retinaldehyde-binding protein. ERK1/2: extracellular signal-regulated kinase1/2. pEKR1/2: phosphorylated ERK1/2. DMSO: dimethyl sulfoxide. CTCF: corrected total cell fluorescence. Scale Bar = 100 µm.

Human retinal explants from the mid-peripheral retina of the same donor were either cultured in Neurobasal-A medium with or without PD98059 for 24 h. We chose 8 µM PD98059 from the *in vitro* studies above as a safe and effective concentration. PD98059 significantly inhibited the phosphorylation of ERK1/2 compared with untreated controls in which it colocalised with the Müller cell marker CRALBP, extending from the OLM to the ILM, assessed by immunostaining (Figure 3A). We also quantified the pERK1/2 staining and CRALBP staining. Manders' coefficient M1 indicated that 80% to 90% pERK1/2 staining colocalised with CRALBP staining in both dimethyl sulfoxide (DMSO, control) and PD98059 treated human retinal explant after 24 h (Figure 3B). Manders' coefficient M2 indicated that more than 80% CRALBP staining colocalised with pERK1/2 staining in DMSO group, but only less than 20% in the PD98059 treatment group, which suggests there is far less pERK1/2 in Müller cells. Quantitative analysis of the corrected total cell fluorescence (CTCF) intensity showed that the PD98059 treatment resulted in significantly lower pERK1/2 expression compared to vehicle alone in 24 h human retinal explants (Figure 3C).

Consistent with the immunostaining findings, PD98059 treatment significantly inhibited the phosphorylation of ERK1/2 (the ratio of pERK1/2 to total ERK1/2) assessed by western blot (Figure 3D, G). The protein levels of total ERK1/2 between the PD98059 group and the DMSO groups were similar (Figure 3E, G). These findings suggest that 8 µM PD98059 effectively inhibits pERK1/2 in *ex vivo* human retinal explants.

### Inhibition of pERK1/2 protects photoreceptors in human retinal explants

**Figure 4:**
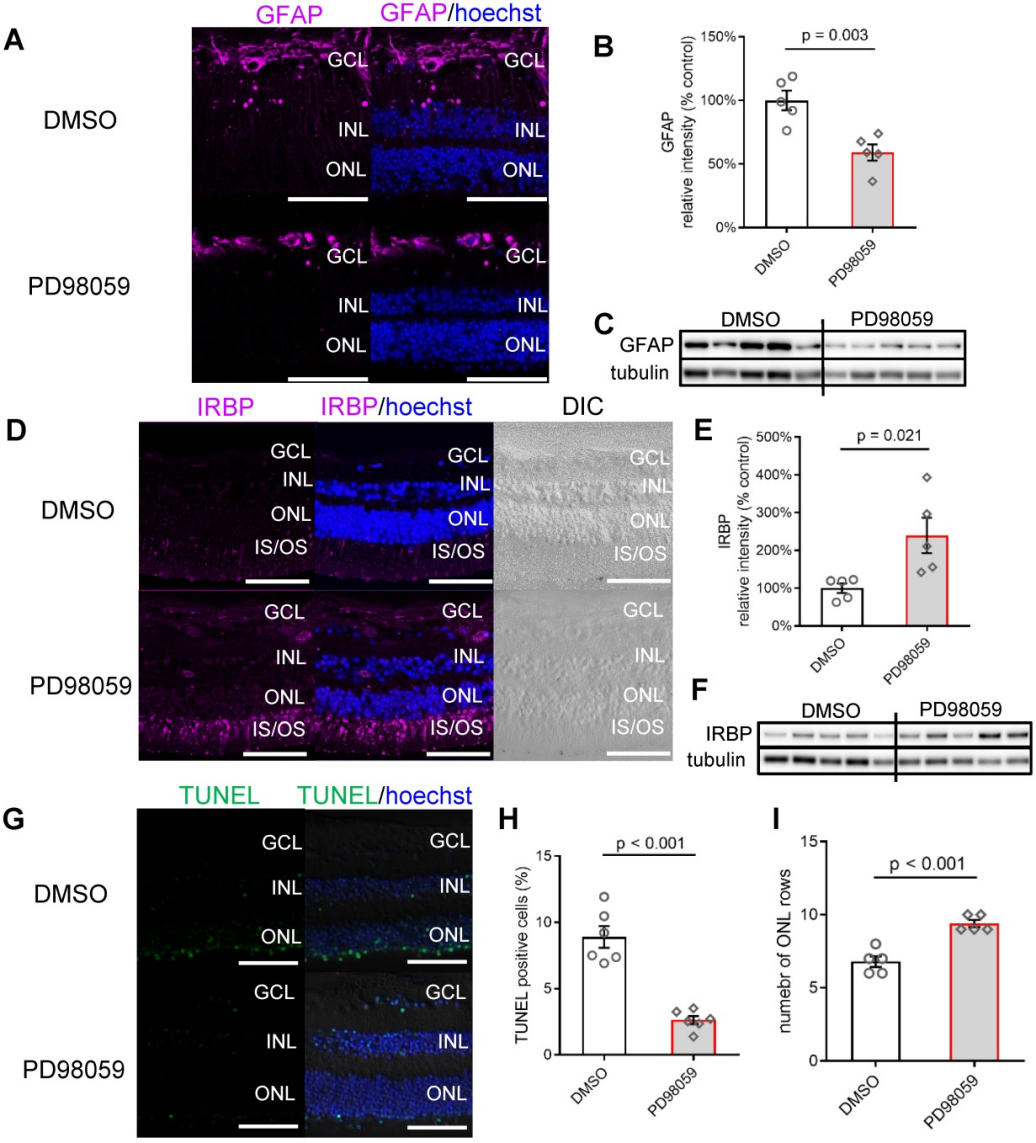
** MAPK (pERK1/2) inhibition protects photoreceptors in human retinal explant, (A)** Vibratome cross-sections of human retinal explants stained for GFAP (magenta) and Hoechst (blue, cell nuclei staining) in DMSO and PD98059 group. **(B, C)** Summary results of western blot results and western blot image of GFAP in human retinal explants (GFAP is normalised by Tubulin) in DMSO and PD98059 group. Data are shown as the mean ± SEM. n = 5. **(D)** Vibratome cross-sections of human retinal explants stained for IRBP (magenta) and Hoechst (blue, cell nuclei staining) in DMSO and PD98059 group. **(E, F)** Summary results of western blot results and western blot image of IRBP in human retinal explants (IRBP is normalised by Tubulin) in DMSO and PD98059 group. Data are shown as the mean ± SEM. n = 5. **(G)** Vibratome cross-sections of human retinal explants stained for TUNEL (green) and Hoechst (blue, cell nuclei staining) in the DMSO and PD98059 group. **(H)** Quantification of TUNEL positive cell ratio in ONL in human retinal explants in DMSO and PD98059 group. Data are shown as the mean ± SEM. n = 6. **(I)** Quantification of photoreceptor cell rows in ONL in human retinal explants in DMSO and PD98059 groups. Data are shown as the mean ± SEM. n = 5. DIC: Differential Interference Contrast. GCL: ganglion cell layer. INL: inner nuclear layer. ONL: outer nuclear layer. IS: inner segments. OS: outer segments. GFAP: glial fibrillary acidic protein. IRBP: interphotoreceptor retinoid-binding protein. DMSO: dimethyl sulfoxide. TUNEL: Terminal deoxynucleotidyl transferase dUTP nick end labelling. Scale Bar = 100 µm.

To assess whether pERK1/2 inhibition in Müller cells affects the response of the human retina to stress, we studied the expression of the glial fibrillary acidic protein (GFAP), a marker of gliosis in Müller cells that indicates the level of retinal stress 49. Immunostaining of GFAP in the inner retina was detected in both 24 h cultured DMSO and PD98059 groups, but the DMSO control retina had intense GFAP immunoreactivity extending into the IPL and INL (Figure 4A), which was significantly reduced by PD98059 treatment.

The western blot (Figure 4B, C) results confirmed the reduction of GFAP at the protein level, which suggest that PD98059 treatment reduces gliosis *ex vivo*.

We then assessed the expression of Interphotoreceptor Retinoid-Binding protein (IRBP), a neuroprotective protein expressed explicitly by photoreceptors 50, in retinal explants cultured for 24 h. We found that IRBP expression, which was seen mainly in the inner segments (IS) and outer segments (OS) of the photoreceptors, was greatly enhanced by treatment with PD98059 (8 µM) (Figure 4D). Western blots found that PD98059 treatment increased IRBP protein levels in the neural retinas (Figure 4E, F). These results suggest that inhibition of pERK1/2 in Müller cells upregulates neuroprotective IRBP expression in photoreceptors.

We also evaluated the protective effect of PD98059 on photoreceptors by terminal deoxynucleotidyl transferase dUTP nick end labelling (TUNEL) staining. Vibratome cross-sections from 24 h cultured human retinal explants with PD98059 (8 µM) treatment appeared to have fewer TUNEL positive photoreceptors than non-treatment control (Figure 4G). Quantification found that TUNEL staining in the ONL was significantly reduced (p < 0.001) after PD98059 treatment (Figure 4H). Explants had more rows of photoreceptor nuclei in their ONLs after PD98059 (p < 0.001, Figure 4I). Taken together, these findings suggest that pERK1/2 inhibition reduces photoreceptor apoptosis in human retinal explants.

### PD98059 inhibited phosphorylation of pERK1/2, predominantly in Müller cells, in photo-oxidative damage mice

**Figure 5:**
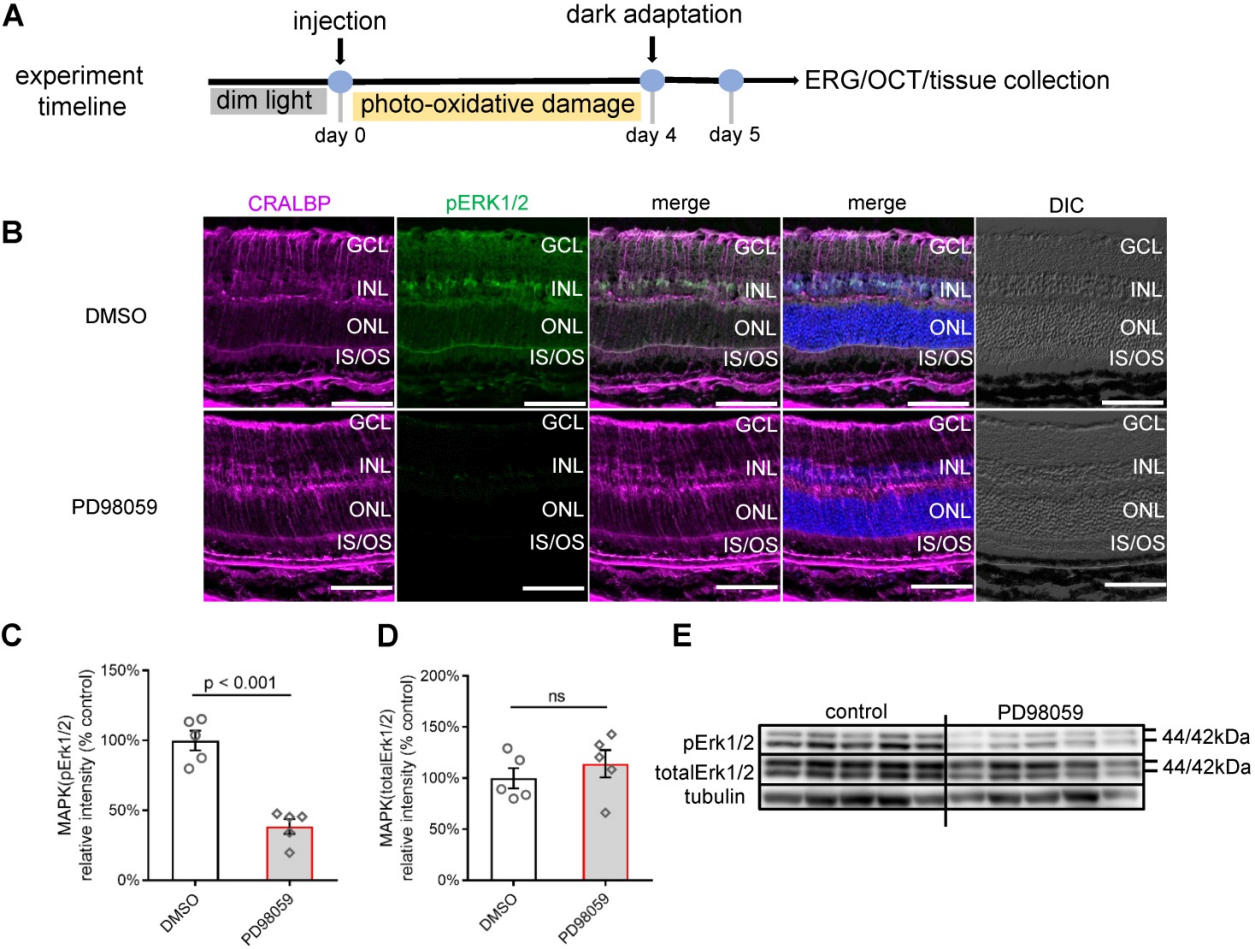
** PD98059 inhibited MAPK (pErk1/2) activation in photo-oxidative damage mice. (A)** Experimental timeline of photo-oxidative damage mice, including injection, dark adaptation, functional assessments (OCT and ERG) and tissue collection. **(B)** Frozen sections of mice retinas stained for CRALBP (magenta), pERK1/2 (green) and Hoechst (blue, cell nuclei staining) in DMSO and PD98059 injected photo-oxidative damage mice eyes. **(C)** Summary results of western blot of pERK1/2 in DMSO and PD98059 injected photo-oxidative damage mice eyes (pERK1/2 is normalised by total ERK1/2). Data are shown as the mean ± SEM. n = 5. **(D)** Summary results of western blot of total ERK1/2 in DMSO and PD98059 injected photo-oxidative damage mice eyes (total ERK1/2 is normalised by Tubulin). Data are shown as the mean ± SEM. n = 5. **(E)** Western blot image of pERK1/2, total ERK1/2 and Tubulin in DMSO and PD98059 injected photo-oxidative damage mice eyes. n = 5. DIC: Differential Interference Contrast. GCL: ganglion cell layer. INL: inner nuclear layer. ONL: outer nuclear layer. IS: inner segments. OS: outer segments. CRALBP: cellular retinaldehyde-binding protein. ERK1/2: Extracellular signal-regulated kinase 1/2. pEKR1/2: phosphorylated ERK1/2. OCT: optical coherence tomography. ERG: Electroretinography. DMSO: dimethyl sulfoxide. Scale Bar = 100 µm.

We next studied whether the protective effects of pERK1/2 inhibition observed *ex vivo* was also seen in an *in vivo* model. We first tested the safety of PD98059 injection in normal mice eyes by TUNEL staining and no apoptotic cells were found (Figure S4). We assessed the effects of PD98059 in mouse retinas in a model using photo-oxidative damage 47. We exposed mice to 100K lux light for 4 days following intravitreal injections of 1 µl of either 100 µM PD98059 or DMSO (Figure 5A). The staining of pERK1/2 colocalised well with the Müller cell marker, CRALBP (Figure 5B). The pERK1/2 was mainly detected in the Müller cell nuclei and in processes extending to the ILM and OLM. The western blot found that PD98059 treatment significantly inhibited the phosphorylation of ERK1/2 compared with the DMSO group (Figure 5C, E), while the protein levels of total ERK1/2 between PD98059 and DMSO groups were similar (Figure 5D, E). We also co-stained pERK1/2 and CRALBP in DMSO injected mice under dim light condition (Figure S5). These data indicate that PD98059 inhibits phosphorylation of ERK1/2 in Müller cells in this murine model of retinal damage.

### pERK1/2 inhibition protected photoreceptors and visual function in photo-oxidative damage mice

**Figure 6:**
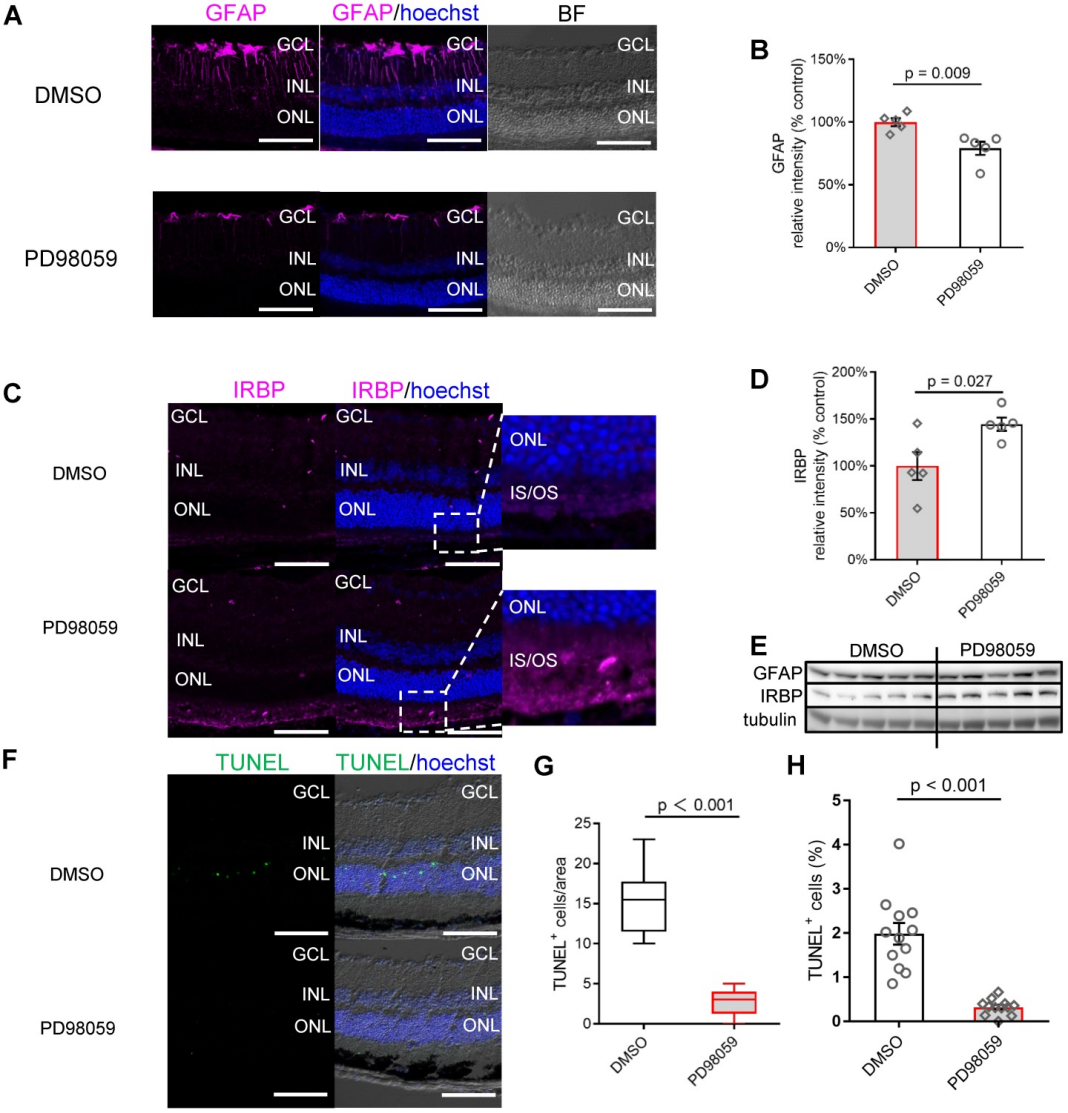
** MAPK (pErk1/2) inhibition protects photoreceptors and visual function in photo-oxidative damage mice. (A)** Frozen sections of mice retinas stained for GFAP (magenta) and Hoechst (blue, cell nuclei staining) in DMSO and PD98059 injected photo-oxidative damage mice. **(B)** Summary results of western blot results of GFAP in DMSO and PD98059 injected photo-oxidative damage mice (IRBP is normalised by Tubulin). Data are shown as the mean ± SEM. n = 5. **(C)** Frozen sections of mice retinas stained for IRBP (magenta) and Hoechst (blue, cell nuclei staining) in DMSO and PD98059 injected photo-oxidative damage mice. **(D)** Summary results of western blot results of IRBP in DMSO and PD98059 injected photo-oxidative damage mice (IRBP is normalised by Tubulin). Data are shown as the mean ± SEM. n = 5. **(E)** Western blot image of GFAP, IRBP and Tubulin in DMSO and PD98059 injected photo-oxidative damage mice eyes. n = 5. **(F)** Frozen sections of mice retinas stained for TUNEL (green) and Hoechst (blue, cell nuclei staining) in DMSO and PD98059 injected photo-oxidative damage mice. **(G)** Quantification of TUNEL positive cell number in DMSO and PD98059 injected photo-oxidative damage mice. Data are shown as the mean ± SEM. n = 12 images from 4 mice. **(H)** Quantification of TUNEL positive cell ratio in DMSO and PD98059 injected photo-oxidative damage mice. Data are shown as the mean ± SEM. n = 12, from 12 different images. DIC: Differential Interference Contrast. GCL: ganglion cell layer. INL: inner nuclear layer. ONL: outer nuclear layer. IS: inner segments. GFAP: glial fibrillary acidic protein. IRBP: interphotoreceptor retinoid-binding protein. DMSO: dimethyl sulfoxide. TUNEL: Terminal deoxynucleotidyl transferase dUTP nick end labelling. Scale Bar = 100 µm.

We next analysed the protective role of PD98059 in the mouse model of retinal damage. We assessed whether the expression of GFAP in mice was similar to that of human retinal explants. The immunofluorescence staining found GFAP expression was also significantly suppressed by PD98059 treatment in mice (Figure 6A). Both DMSO and PD98059 groups had GFAP immunolabelling in the superficial retina, indicating the expression in the astrocytes 51. Photo-oxidative damaged retinas additionally had intense GFAP immunoreactivity in Müller cells throughout the entire cell (Figure 6A), which was significantly reduced by inhibition of pERK1/2 (Figure 6A). Consistent with the immunostaining studies, western blot results found PD98059 treatment significantly inhibited the expression of GFAP protein (Figure 6B, E). These results are consistent with our findings in human retinal explants, indicating that inhibition of pERK1/2 by PD98059 relieves retinal stress.

The immunostaining found that PD98059 treatment also significantly increased the expression of neuro-protective IRBP in photoreceptors which is concentrated at the photoreceptor OS (Figure 6C). Western blot quantification verified the upregulation of IRBP at the protein level in the group treated with PD98059 (Figure 6D, E). The PD98059 treatment group appeared to have fewer apoptotic photoreceptors (TUNEL positive, green) in the ONL compared to the control group (Figure 6F). Confocal image analysis showed that PD98059 treatment significantly reduced the TUNEL positive cells in ONL (Figure 6G, H).

**Figure 7:**
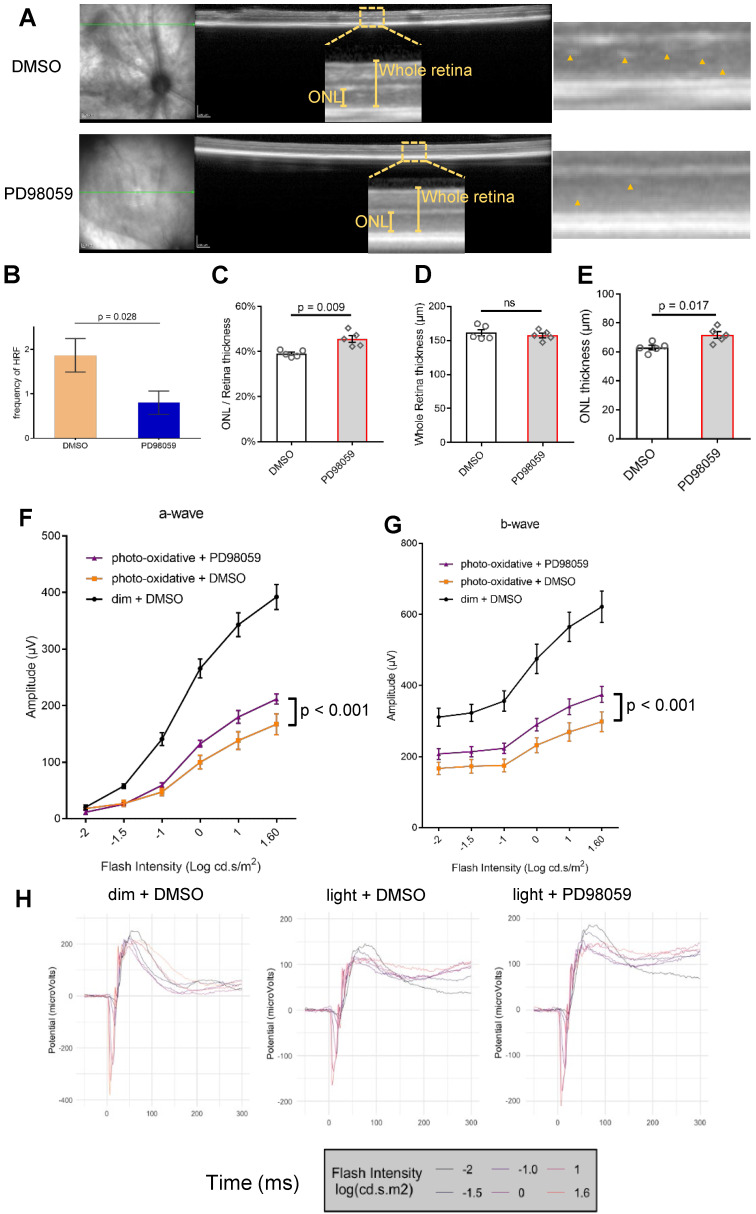
** MAPK (pERK1/2) inhibition protects photoreceptors and visual function in photo-oxidative damage mice. (A)** Representative OCT images in DMSO and PD98059 injected photo-oxidative damage mice and blow-ups. Yellow arrowheads indicate the HRF of ONL. **(B)** Quantification of HRF of ONL in OCT of DMSO and PD98059 injected photo-oxidative damage mice (P < 0.001). Data are shown as the mean ± SEM. n = 5. **(C-E)** Quantification of (D) whole retina thickness, (E) ONL thickness and (C) their ratio in DMSO and PD98059 injected photo-oxidative damage mice. Data are shown as the mean ± SEM. n = 5. There was minimal effect on whole retina thickness in PD98059 injected photo-oxidative damage mice compared with DMSO control. There was a significant increase in photoreceptors (ONL thickness) in PD98059 injected photo-oxidative damage mice compared with DMSO control (P < 0.05). There was a significant increase in photoreceptors (ratio of ONL thickness to whole retina thickness) in PD98059 injected photo-oxidative damaged mice compared with DMSO control (P < 0.05). **(F, G)** Retinal function was measured in DMSO and PD98059 injected photo-oxidative mice eyes or dim light control mice using ERG. Photo-oxidative damage mice had a significantly lower retinal function compared to dim light controls, for both (F) a-wave and (G) b-wave (P < 0.05, Dim + DMSO group, n = 5; Light + DMSO group, n = 6; Light + PD98059 group, n = 6). However, in PD98059 injected mice, retinal function was significantly higher than DMSO injected mice when exposed to photo-oxidative stress for both (F) a-wave and (G) b-wave. P < 0.05, Light + DMSO group, n = 6; Light + PD98059 group, n = 6. Data are shown as the mean ± SEM. **(H)** Mean ERG traces in DMSO and PD98059 injected photo-oxidative mice eyes or dim light control mice. Light + DMSO group, n = 6; Light + PD98059 group, n = 6). ONL: outer nuclear layer. HRF: hyperreflective foci. DMSO: dimethyl sulfoxide.

We further explored the effect of PD98059 treatment on morphologic features of the photo-oxidative damaged retina by *in vivo* optical coherence tomography (OCT) (Figure 7A and Figure S6). We quantified the frequency of hyperreflective foci (HRF), which represent a consensus designation for age-related macular degeneration (AMD) 52. We found PD98059 significantly reduced these HRF in ONL in the photo-oxidative mice model (Figure 7B). We also measured the retinal ONL thickness using ImageJ. We found that whole retinal thicknesses were similar (Figure 7D), but the thickness of ONL and its ratio to the thickness of the whole retina was significantly greater in the group that received PD98059 treatment (Figure 7C, E). These data suggest that PD98059 reduces photoreceptor apoptosis and protects photoreceptors from degeneration *in vivo*.

We evaluated the protective effects of PD98059 treatment on retinal function under photo-oxidative stress using electroretinography (ERG) (Figure 7F-H). Photo-oxidative stressed mice had significantly reduced a-wave and b-wave amplitudes compared to dim light controls (Figure 7F, G). Treatment with PD98059 significantly restored the reduced a-wave and b-wave amplitudes in the mice exposed to photo-oxidative stress (Figure 7F, G).

### Crosstalk between Müller cells and photoreceptors involved JAK/STAT3 signalling pathway in human retinal explants

We performed next-generation sequencing to understand the retinal signalling changes after pERK1/2 inhibition at a transcriptional level. Retinal tissues from 4 different human eye donors were cultured for 24 h with PD98059 or the same concentration of DMSO, after which total RNA was extracted and its quality assessed. Next-generation sequencing was performed on eight samples in total (4 human donors, PD98059 *versus* DMSO control groups) (Table 1). An initial 35,386 genes, including both protein-coding genes and non-coding genes, were filtered for quality, SNPs and functional relevance, leading to 363 (1.03%) genes found to be differentially expressed.

**Figure 8:**
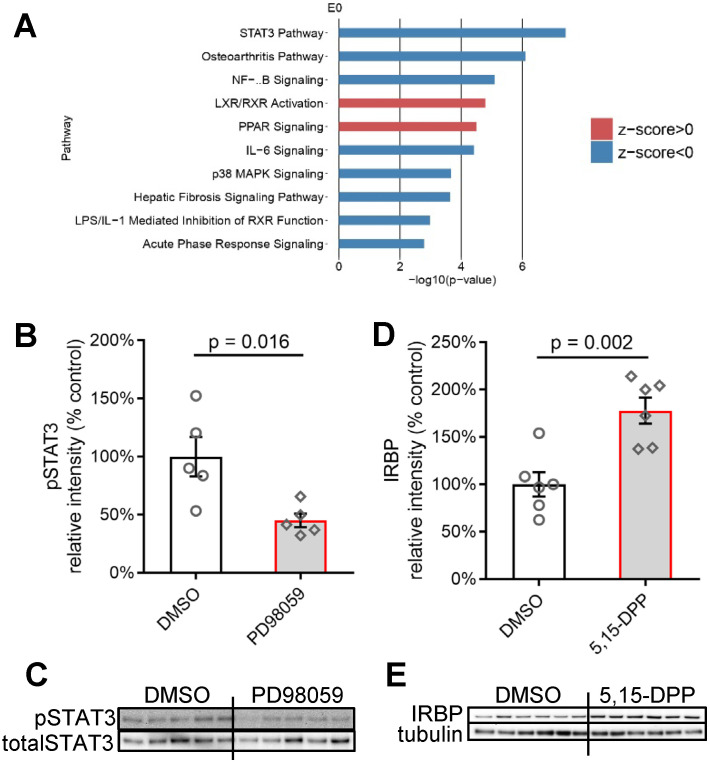
** Crosstalk between Müller cells and photoreceptors involved Jak/STAT3 signalling pathway. (A)** IPA of top 10 changed canonical pathways in MAPK inhibition human retinal explants compared with control human retinal explants after 24 h culture. *Blue bars*: negative z-score; *red bars*: positive z-score. n = 4. **(B)** Summary results of western blot of pSTAT3 in DMSO or PD98059 treated human retinal explants after 24 h culture (pSTAT3 is normalised by totalSTAT3). Data are shown as the mean ± SEM. n = 5. **(C)** Western blot image of pSTAT3 and totalSTAT3 in DMSO or PD98059 treated human retinal explants after 24 h culture. n = 5. **(D)** Summary results of western blot of IRBP in DMSO or 5,15-DPP (STAT3 inhibitor) treated human retinal explants after 24 h culture (IRBP is normalised by Tubulin). Data are shown as the mean ± SEM. n = 6. **(E)** Western blot image of IRBP and Tubulin in DMSO or 5,15-DPP treated human retinal explants after 24 h culture. IRBP: interphotoreceptor retinoid-binding protein. STAT3: signal transducer and activator of transcription 3. pSTAT3: phosphorylation of STAT3. IL-1 beta: interleukin-1 beta. DMSO: dimethyl sulfoxide. RNA: ribonucleic acid. IPA: Ingenuity Pathway Analysis. 5,15-DPP: 5,15-Diphenylporphyrin.

We performed QIAGEN Ingenuity Pathway Analysis (IPA) on all the differentially expressed genes (p < 0.05) to identify canonical pathways that significantly changed after inhibition of MAPK (ERK1/2) signalling (Figure 8A) in the 24 h cultured human retina. Among the top 15 changed canonical pathways (*Blue bars*: negative z-score; *orange bars*: positive z-score) after pERK1/2 inhibition (p < 0.001), the JAK/STAT3 signalling pathway was most greatly inhibited (-log(p-value) = 7.42).

Western blotting did confirm that PD98059 treatment significantly inhibited the phosphorylation levels of STAT3 (the ratio of phosphorylated STAT3 to total STAT3) (Figure 8B, C) in the 24 h cultured human retina. We further tried directly inhibiting the JAK/STAT3 signalling in human retinal explants using a JAK/STAT3 inhibitor 5,15-Diphenylporphyrin (5,15-DPP) and found the treatment increased the expression of IRBP in photoreceptors (Figure 8D, E), which is consistent to the response of photoreceptors under pERK1/2 inhibition.

We also checked the transcriptional expression of cytokines such as fibroblast growth factor 2 (FGF2), CNTF, IL-1 beta, LIF and their corresponding receptors fibroblast growth factor receptor 2 (FGFR2), ciliary neurotrophic factor receptor (CNTFR), interleukin 1 receptor type 1 (IL-1R1), leukemia inhibitory factor receptor (LIFR) in our RNA sequencing data from the DMSO or PD98059 treated human retinal explants (Figure S7). IL-1 beta, IL-1R1 and LIF are significantly downregulated in PD98059 treated human retinal explants, while there were no significant changes in the transcription levels of FGF2, FGFR2, CNTF, CNTFR and LIFR (Figure S7).

## Discussion

The response of Müller cells to retinal stress is critical in maintaining retinal homeostasis. The molecular signalling between Müller cells and photoreceptors under stress and its contribution to the pathogenesis of retinal diseases is not fully understood. Distinguishing roles for certain signalling pathways in retinal diseases may provide new avenues for treatment. We found that pERK1/2 was mainly activated in Müller cells in stressed retinas *ex vivo* and *in vivo* and provided evidence that targeted blocking of MAPK (ERK1/2) activation in Müller cells may protect photoreceptors. Our findings elucidate the intimate connection between Müller cells and photoreceptors. Transcriptomic analyses also indicated that the JAK/STAT3 signalling pathway might mediate the transduction of signals from Müller cells to photoreceptors.

This study utilised both *ex vivo* human retinal explants and an *in vivo* photo-oxidative damage murine model to study the effects of pERK1/2 inhibition in the stressed retina. *Ex vivo* human retinal explants retain the complex cell-to-cell interactions in the retina. Therefore, they are considered a more appropriate model for translational research on the retina than, for example, monoculture or coculture systems 53. Photo-oxidative damage, which induces photoreceptor death, is a good model to study the effects of retinal oxidative stress and photoreceptor loss 47. This study observed that pERK1/2 inhibition was associated with reduced photoreceptor degeneration in both *ex vivo* human retinal explants and the *in vivo* photo-oxidative damage model.

We observed that pERK1/2 was activated mainly in Müller cells under stress in *ex vivo* human retinal explants, in murine retinas damaged by photo-oxidation and in a human eye with late-stage GA. This is consistent with previous reports of the activation of ERK1/2 signalling in glial cells in diseased animals 41, 44, 45, 54-56. This suggests that activation of pERK1/2 in Müller cells may be a typical response to retinal stress and may be a feature of other degenerative retinal diseases.

PD98059, which inhibits the phosphorylation of ERK1/2 by binding to inactive forms of MAPK (ERK1/2) kinase 38, 39, has shown promising therapeutic effects in several models of retinal disease. Intravitreal administration of PD98059 has been reported to inhibit the pERK1/2 and prevent degeneration in Dicer1 gene knockout mice or reduce the area of retinal neovascularisation in a rat model of retinopathy prematurity 57, 58. However, how PD98059 acts within the retina is poorly understood. The present study found that PD98059 inhibited the phosphorylation of ERK1/2 mainly in stressed Müller cells, leading to downstream changes in the photoreceptors. The limitation of the specificity of using the small molecule inhibitors is worth mentioning. Although there is no report about the additional target of the PD98059, we cannot exclude the possibility that the inhibitor affects additional pathways, contributing to the phenotype we observed. For example, even though ERK1/2 is not obviously activated in photoreceptors, PD98059 may still have off target direct effects on them.

We focused on two major disease-related proteins to evaluate the protective effects of MAPK (ERK1/2) inhibition: GFAP, a specific marker of Müller cell gliosis under retinal stress 1 and the photoreceptor-specific marker IRBP, which response to the dysfunction of Müller cells 23.

The role of GFAP activation in response to retinal stress remains controversial. It has been reported that reduced GFAP expression increased photoreceptor degeneration 59, while other studies found GFAP activation in Müller cells correlated to retinal degeneration 47. As a typical stress marker specifically expressed in Müller cells, the GFAP activation represents a Müller cells response to retinal insult. In the short term, this response may be protective, but long term may be detrimental. In this study, the inhibition of GFAP observed after the PD98059 treatment suggests relieved retinal stress.

IRBP is an essential photoreceptor-specific protein responsible for retinoid transport between the RPE and photoreceptors in the eye. The antioxidant activity of IRBP is essential in maintaining the redox environment of the subretinal space 60. Functional IRBP is critical for the survival of photoreceptors and vision 50. Several studies have suggested that the downregulation of IRBP is an early sign of photoreceptor degeneration 50, 61, 62. In turn, IRBP upregulation has been reported to delay photoreceptor degeneration in diabetic mice and rats 63, 64. The restoration of IRBP expression after pERK1/2 inhibition in the present study was associated with improved photoreceptor viability and function in response to the various stresses.

This study used OCT to assess HRF and the thickness of the ONL in photo-oxidative stressed mice. HRF, which were observed in the ONL of light stressed mouse retinas, were significantly reduced by the treatment of PD98059. HRF is a consensus designation for interpreting OCT scans from eyes with AMD 52. HRF in patients with AMD is thought to represent RPE cells that migrate inwardly to approach the retinal vessels and lipid-filled cells that may be microglia 65-68. The reduction in numbers of HRF and reduced thinning of the ONL in PD98059 treated eyes support the hypothesis that pERK1/2 inhibition prevents photoreceptor degeneration induced by photo-oxidative stress. The light stress mice model is an acute photoreceptor degeneration model involving multiple adverse factors that induce retinal degeneration 47. The interference of one signalling pathway, MAPK (ERK1/2) signalling, may have a protective effect on photoreceptors but is probably not potent enough to reverse the entire stressful environment in the retina.

The IPA analysis based on RNA transcriptomic results revealed potential signalling cascades between Müller cells and photoreceptors under stress. We identified that JAK/STAT3 signalling might be necessary to pass the signal from stressed Müller cells to photoreceptors. Activation of JAK/STAT3 signalling may play different roles at different stages of the disease. Some may be protective 69, while others have reported destructive activity 70, 71. In this study, directly inhibiting JAK/STAT3 signalling had the same protective effect (IRBP upregulation) on photoreceptors as the inhibition of MAPK (ERK1/2) signalling in Müller cells. This observation indicates that the JAK/STAT3 signalling may be downstream of MAPK (ERK1/2) signalling that is involved in the response of photoreceptors in retinal degeneration. It should also be noted that the JAK/STAT3 signalling was activated in degenerative photoreceptors but not confined to photoreceptors 29, 72.

We also identified significant transcriptional changes in levels of of IL-1 beta and LIF, both of which are reported to be secreted by Müller cells 25, 26. Both IL-1 beta and LIF can be induced by ERK1/2 signalling and directly activate JAK/STAT3 signalling 30-32, 73-78. These two cytokines may contribute to the communication between Müller cells and other retinal cell types, including photoreceptors. The detailed roles of IL-1 beta and LIF linked between these two signalling pathways within different retinal cell types warrant further investigation.

This study describes how the activation of MAPK (ERK1/2) signalling, mostly in Müller cells, may affect photoreceptors, thus revealing a basis for the intimate interaction between Müller cells and photoreceptors during retinal degeneration. It may be a potential therapeutic strategy targeting such signalling pathways in Müller cells to treat retinal diseases with underlying photoreceptor degeneration such as AMD and DR.

## Methods

### Cell Culture

The MIO-M1 cell line was obtained from the UCL Institute of Ophthalmology, London, UK 79 and cultured in DMEM (Gibco, Amarillo, TX, 10569) with 10% fetal bovine serum (FBS) (Sigma‐Aldrich, Burlington, MA, F9423) and 1% penicillin/streptomycin (Sigma‐Aldrich, Burlington, MA, P4333) in a humidified 5% CO_2_ incubator at 37 °C. HuPMCs were prepared and obtained from *post-mortem* donor eyes as described previously 80-82. HuPMCs culture conditions were the same as the MIO-M1 cell line. The Y79 retinoblastoma cell line (ATCC Cat# HTB‐18, RRID:CVCL_1893) was cultured in RPMI-1640 medium (Sigma‐Aldrich, Burlington, MA, R8758) with 10% FBS and 1% penicillin/streptomycin in a humidified 5% CO_2_ incubator at 37 °C.

### PD98059 and 5,15-DPP

PD98059 (Sigma‐Aldrich, Burlington, MA, P215) was dissolved in DMSO (Sigma‐Aldrich, Burlington, MA, C6295) with a 20mM stock concentration. 5,15-DPP (Sigma‐Aldrich, Burlington, MA, D4071) was dissolved in DMSO with a 5mM stock concentration. The stock solution was diluted in a culture medium for *in vitro*/*ex vivo* culture or phosphate-buffered saline (PBS) for intravitreal injection.

### Lactate Dehydrogenase (LDH) Cytotoxicity Assay and AlamarBlue Cell Viability Assay

MIO-M1 cells and huPMC cells with 70% confluence were pretreated in a DMEM starvation medium overnight in a 96‐well plate (Corning, NY, 3340). Y79 cells were pretreated in RPMI 1640 starvation medium overnight. The following day, cells were centrifuged and resuspended in media (1×10^5^ cells/ml) with or without different concentrations of PD98059 in a 96‐well plate. The following day, the culture medium was changed to medium with or without different concentrations of PD98059. After 22 h treatment, 15 μl of supernatant from every well was transferred into a 384‐well plate (Corning, NY, 3680) for the LDH cytotoxicity assay (Thermo Fisher Scientific, Waltham, MA, 88954). The reaction was mixed according to the manufacturer's protocol. The absorbance of each well was measured at 480nm and 680nm by a safire2 plate reader (Tecan, Männedorf, Switzerland). Cell metabolic rate was measured by AlamarBlue cell viability assay with 100 μl of the medium in each well of the 96‐well plate supplemented with 10 μl of AlamarBlue cell viability reagent (Thermo Fisher Scientific, Waltham, MA, DAL1100) and incubated at 37 °C for 1 h. The fluorescence was detected by a plate reader. All data were normalised to the DMSO control group.

### Western Blot

Different samples for western blot (including cells, human retinal explants or mice retinas) were lysed in RIPA buffer with a 1% protease/phosphatase inhibitor cocktail (Cell Signalling Technology, Danvers, MA, 5872S), centrifuged at 12,000× g at 4 °C for 10 m and the supernatant was collected. Protein concentration measurement was conducted with a bicinchoninic acid protein assay (Sigma‐Aldrich, Burlington, MA, BCA1-1KT). Cell lysates with the same amount were heated for 10 m at 70 °C, adding NuPAGE loading dye and reducing buffer (Life Technologies, Carlsbad, CA). After loading onto NuPAGE 4-12% Bis-Tris Protein gels (Thermo Fisher Scientific, Waltham, MA), the samples were electrophoresed at 180 V, 4 °C for 70 m. The proteins were transferred to PVDF membranes (Millipore, Burlington, MA) using the iBlot gel transfer device (Thermo Fisher Scientific, Waltham, MA). The PVDF membranes were blocked with 5% bovine serum albumin, Tris-Buffered Saline and 0.1% Tween 20 for 1 hr at room temperature and then incubated with primary antibody overnight at 4°C and secondary antibody for 2 h at room temperature. Antibodies used, including their company, species, catalogue number and working dilution, are listed in Table 2. Protein expression was visualised using ECL (Bio‐rad, Hercules, CA, 170-5061) and quantified with the Gene Tools image scanning and analysis package.

### *Ex vivo* human retinal explant culture

Human neural retinas were obtained from *post-mortem* healthy donor eyes within 24 h of death. Mid‐peripheral regions were trephined with a 3mm diameter biopsy punch (Kai Medical, Japan). The sample was then transferred to a 12‐well Transwell plate (Corning, NY, 3460) and cultured in Neurobasal-A medium (Thermo Fisher Scientific, 21103049) supplemented with 1% FBS, 2% B‐27 (Gibco, Amarillo, TX, 17504), 1% N‐2 (Gibco, Amarillo, TX, 17502), 1% Glutamax (Gibco, Amarillo, TX, 35050), 1% ITS-X (Gibco, Amarillo, TX, 51500) and 1% penicillin-streptomycin , with or without 8 μM PD98059, respectively. Retinal explants were incubated at 37 °C in 5% CO_2_ for 24 h with the photoreceptor layer facing up.

### Vibratome sectioning and Immunofluorescence staining

4% paraformaldehyde (PFA) in PBS buffer was used to fix the 3mm diameter retinal explants for 1 h. Following this, retinas were transferred to fresh PBS and embedded in 3% low‐melting Sea Plaque Agarose (Lonza, Rockland, ME, 50100) in PBS. The embedding blocks were sectioned at a 100 μm thickness using a vibratome (Leica, Germany, VT1200S) and stored in 48‐well plates (Corning, NY, 3548) with PBS at 4 °C until use. Tissue sections were blocked in 5% donkey serum and 1% Triton‐100 in PBS in 48‐well plates overnight. The primary antibody was diluted in PBS supplemented with 1% Donkey Serum and 1% Triton-100 for 4 days at 4 °C. The secondary antibody was diluted in PBS supplemented with 1% Donkey Serum for 2 days at 4 °C. Nuclei were stained with Hoechst 33342 at room temperature for 30 m. Antibodies used, including their company, species, catalogue number and working dilution, are listed in Table 2. Vibratome cross-sections were washed three times with PBS and mounted onto polylysine glass slides (Thermo Fisher Scientific, Waltham, MA) using Vectorshield Antifade Mounting Medium (Vector Laboratories, Burlingame, CA, H-1000) and coverslips.

### Paraffin sectioning and Immunofluorescence staining

Paraffin sections of the human GA retina were obtained as described previously 83. Eye tissues were sectioned at a 6 μm thickness using a microtome (Leica, Germany) and stored at room temperature. Paraffin sections are deparaffinised with xylene 2 times, 10 m each and rehydrated in a graded series of ethanol (100%, 90%, 70%, 50%, PBS, 5 m each). After heating slides in 10mM sodium citrate buffer (PH = 6.0) to 100 °C for 20 m, slides were cooled down and transferred to PBS. Following dewax, hydration and antigen retrieval, slides were blocked in 5% normal donkey serum for 30 m at room temperature. Overnight primary antibody incubation and 4 h secondary antibody incubation were then conducted in the presence of 1% normal donkey serum and 0.1% Triton-100. Nuclei were stained with Hoechst 33342 at room temperature for 5 m. Tissue-Tek O.C.T. Compound and coverslips were used to mount the slides.

### Microscopy

Fluorescence was detected using a confocal microscope (Zeiss, Germany, LSM 700). High-resolution (2048 ×2048 pixels) confocal images were taken with a 20× (Plan Apochromat no. 420650-9901) objective. Zen Software, Blue Edition (ZEN Digital Imaging for Light Microscopy from Zeiss, Germany, RRID:SCR_013672) was used to adjust their contrast and brightness.

### Photo-oxidative damage mouse

All procedures were performed following the Association for Research in Vision and Ophthalmology Statement for the Use of Animals in Ophthalmic and Vision Research. C57BL/6J mice were born and raised in dim (5 lux) cyclic light conditions in a 12:12 h cycle and housed in individually ventilated cages. Animals had free access to food and water. Six-week adult mice were used for all experiments. C57BL/6J mice were intravitreally injected with 1 μl 100 μM PD98059 or the same concentration solvent, 0.5% DMSO, in both eyes for the treatment experiment. Then, mice were continuously irradiated with a 100K lux white cold light source for 4 days to establish a photo-oxidative retinal degeneration model 47. We also intravitreally injected C57BL/6J mice with 1 μl 0.5% DMSO and raised in dim cyclic light conditions in a 12:12 h cycle as dim light controls. Pupil dilation was performed twice daily (in the morning and evening). Animals were euthanised with CO_2_ prior to tissue collection. The left eye from each animal was marked for orientation and then enucleated for cryosectioning, while the retina from the right eye was collected for protein extraction.

### Optical Coherence Tomography (OCT)

A Spectralis HRA+OCT device (Heidelberg Engineering, Heidelberg, Germany) was used to take the fundus and cross-sectional images of live mice. We anesthetised mice by intraperitoneal injection of Ketamine (100 mg/kg; Troy Laboratories, NSW, Australia), and Xylazil (12 mg/kg; Troy Laboratories). Mice pupils were dilated with Tropicamide 0.5% eye-drops (Bausch and Lomb, Tampa, USA). Mice were restrained on a custom-made platform attached to the imaging device. Hypromellose 0.3% eye drops (GenTeal; Novartis, NSW, Australia) were administered to maintain corneal hydration and improve OCT image quality. We placed a rodent contact lens on the mice cornea (PMMA lenses, the radius of curvature of the central optic zone of 2.70 mm and diameter of 5.20 mm; Cantor + Nissel, Brackley, UK). We captured fundus and cross-sectional images from 0 to 3 mm superior to the optic nerve (ON) and 1 to 2 mm inferior to the ON. We placed Eye gel (GenTeal; Novartis) on both eyes for recovery after measurement.

Hyperreflective dots on OCT were counted by an experienced retinal specialist who was masked to the experimental conditions. Three equidistance zones 400µm wide from whole retinal scans were graded. A single value for each OCT scan was obtained by summing the numbers from the three zones of interest.

We measured retinal thickness on the cross-sectional images around 1 mm superior to ON. Five points were measured to get the average thickness. We defined the ONL thickness ratio as the ratio between ONL thickness and the distance between the OLM and ILM, as described previously 84.

### Electroretinography (ERG)

We performed ERG to evaluate retinal function in response to full-field flash stimuli under scotopic conditions. Animals were injected with an inhibitor or DMSO and exposed to light stress for 4 days. They were then removed from the light, dark-adapted overnight, anaesthetised and placed on the ERG ganzfeld. Then an LED-based system (FS-250 A Enhanced Ganzfeld, Photometric Solutions International, Melbourne) provided flash stimuli over a stimulus intensity range of 3.9 log cd s m^-2^ (range -2 to 1.9 log cd s m^-2^). Data extraction and analysis for both a-wave and b-wave responses were performed as previously described 47, showing the mean wave amplitude (uV) for each flash intensity.

### Cryosectioning and immunofluorescence staining

Mice's eyes were immediately immersed in 4% PFA for 3 h at room temperature. Following this, eyes were washed with fresh PBS twice and equilibrated in 30% sucrose overnight. Embedding in Tissue-Tek O.C.T. Compound, the blocks were sectioned at a 10 μm thickness using a Cryostat (LEICA CM3050S, Germany), attached to Cryostat slides and stored at -20 °C until use. Cryosections were circled with a wax pen and blocked in 10% donkey serum for 1 h. The primary antibody was diluted in PBS supplemented with 5% Donkey Serum and 0.3% Triton-100 overnight at 4 °C. The secondary antibody was diluted in PBS supplemented with 1% Donkey Serum 1% Triton-100 for 2 h at room temperature. Nuclei were stained with Hoechst 33342 at room temperature for 5 m. Cryosections were washed 3 times with PBS. Tissue-Tek O.C.T. Compound and coverslips were used to mount the slides. Fluorescence was detected using Zeiss confocal microscope, and Zen Software, Blue Edition was used to adjust their contrast and brightness.

### Fluorescence Intensity and Colocalisation Analysis

Fluorescence intensity of pERK1/2 was quantified by the FIJI version of ImageJ (ImageJ 1.53q) software (https://imagej.net/software/fiji/downloads, National Institutes of Health, USA). Area, integrated density were measured and background areas were used to normalise against autofluorescence. The CTCF was calculated using the formula: CTCF = Integrated Density - (Area of quantification X Mean fluorescence of background readings).

Colocalisation analysis was performed using the JACoP plugin in the FIJI version of ImageJ (ImageJ 1.53q) software (https://imagej.net/software/fiji/downloads, National Institutes of Health, USA). Manders' correlation coefficients (M1 and M2) were used to predict the co-occurrence of the fluorescence channels. M1 indicates the overlap of the pERK1/2 signal with the CRALBP signal and M2 indicates the overlap of the CRALBP signal with the pERK1/2 signal.

### Terminal deoxynucleotidyl transferase dUTP nick end labelling (TUNEL) staining and quantification

Retinal cryosections and vibratome sections were stained for apoptotic cells using a TUNEL kit (Roche, Switzerland, 11684795910). Sections were incubated in the reaction solution in a humid chamber at 37 °C for 1 h. Representative cryosection and vibratome cross-section images of 10.24 mm^2^ were taken from the superior retina, approximately 500 µm from the optical nerve, to quantify photoreceptor cell death. We used ImageJ to count all the TUNEL positive cells and all the Hoechst positive staining nuclei (including INL and ganglion cell layer, GCL). The ratio of the two was defined as the TUNEL positive ratio. Three technical duplicates were counted and averaged for each section of each experimental group.

### RNA sequencing and analysis

Total RNA from the cultured human retinal explants, with or without PD98059 treatment (n = 4), was extracted using GenElute™ Single Cell RNA Purification Kit (Sigma Aldrich, Burlington, MA, RNB300). mRNA was enriched using oligo magnetic beads. The library preparation, sequencing and quality control were commercially contracted to BGI (https://www.bgi.com/global/). Briefly, mRNA was fragmented into short fragments (200 ~ 700 bp) in the fragmentation buffer, and first-strand cDNA was synthesised with random hexamer primers using the mRNA fragments as templates, followed by the second-strand synthesis. The double-stranded cDNA was purified with a QiaQuick PCR extraction kit and then used for end repair and base A addition. Finally, sequencing adapters were ligated to the fragments purified by SPRI bead size selection and enriched by PCR amplification. The library products were sequenced using Illumina HiSeq 2500 with paired-end 100 bp read length.

RNA sequencing data are available at https://www.ncbi.nlm.nih.gov/geo/query/acc.cgi?acc=GSE205370.

The University of Sydney provided the Ingenuity Pathway Analysis software license from Qiagen (QIAGEN Inc., https://www.qiagenbioinformatics.com/products).

### Statistics

All data are expressed as mean ± SEM. SPSS version 17.0 for Windows software was used to perform the statistical analyses. One-way ANOVA was used to compare differences between mean values of three or more groups throughout the study. The differences between mean values of any given two groups throughout the study were evaluated using Student's t-tests. A statistically significant difference was considered when the p-value was less than 0.05.

### Study approval

The protocols for human retina studies were approved by the Human Research Ethics Committee of the University of Sydney (HREC#: 2016/282). The protocols for animal studies were approved by the Australian National University Animal Experimentation Ethics Committee (Ethics ID: A2017/41).

## Supplementary Material

Supplementary figures.

## Figures and Tables

**Table 1:** Donor information for human retinal explants

Donor ID	Gender	Age	Known eye disease
200169	Female	72y	No
200230	Female	53y	No
200590	Male	71y	No
201258	Female	79y	No

**Table 2:** List of antibodies successfully used in this study

Molecular Marker (initials)	Company	Source	Catalog No.	Working dilution	
				WB	IF
pERK1/2	cell signalling	Rabbit	CS4370	1:1000	1:200
ERK1/2	cell signalling	Rabbit	CS9102	1:1000	N/A
pSTAT3	cell signalling	Rabbit	CS9145	1:1000	N/A
Stat3	cell signalling	Rabbit	CS4904	1:1000	N/A
CRALBP	Abcam	Mouse	AB15051	1:1000	1:500
GS	Millipore	Mouse	MAB302	N/A	1:200
GFAP	Millipore	Mouse	MAB360	1:1000	1:500
IRBP	Santa Cruze	Mouse	SC390218	1:1000	1:100
α/β-Tubulin	cell signalling	Rabbit	CS2148	1:1000	N/A
Anti-rabbit IgG	cell signalling	Goat	CS7074	1:5000	N/A
Anti-mouse IgG	cell signalling	Horse	CS7076	1:5000	N/A
Anti-mouse-594	Invitrogen	Donkey	A21203	N/A	1:5000
Anti-rabbit-488	Invitrogen	Donkey	A21206	N/A	1:5000
Hoechst 33342	Thermo Fisher		H3569	N/A	1 µg/ml

## Abbreviations

CTCF: corrected total cell fluorescence
DIC: differential interference contrast
DMSO: dimethyl sulfoxide
ERG: electroretinography
ERK1/2: extracellular signal-regulated kinase 1/2
FGF2: fibroblast growth factor 2
FGFR2: fibroblast growth factor receptor 2
GA: geographic atrophy
GCL: ganglion cell layer
GFAP: glial fibrillary acidic protein
GS: glutamine synthetase
HRF: hyperreflective foci
huPMCs: human primary Müller cells
IL-1 beta: interleukin-1 beta
IL-6: interleukin-6
ILM: inner limiting membranes
INL: inner nuclear layer
IPA: Ingenuity Pathway Analysis
IPL: inner plexiform layer
IRBP: interphotoreceptor retinoid-binding protein
IS: inner segments
JAK/STAT3: janus kinase /signal transducer and activator of transcription 3
LDH: lactate dehydrogenase
LIF: leukemia inhibitory factor
LIFR: leukemia inhibitory factor receptor
MAPK: mitogen-activated protein kinase
MIO-M1: Moorfields/Institute of Ophthalmology-Müller 1
OCT: optical coherence tomography
OLM: outer limiting membrane
ONL: outer nuclear layer
OS: outer segments
PBS: phosphate-buffered saline
pERK1/2: phosphorylated ERK1/2
PFA: paraformaldehyde
RPE: retinal pigment epithelium
TUNEL: terminal deoxynucleotidyl transferase dUTP nick end labelling
