# Supplementary material for: Inhibiting the activation of MAPK (ERK1/2) in stressed Müller cells prevents photoreceptor degeneration
Source: Theranostics. 2022 Sep 11;12(15):6705–22. doi: 10.7150/thno.71038 (PMC9516241; doi:10.7150/thno.71038)
Supplement: Supplementary file 1 — Supplementary figures. [file thnov12p6705s1.pdf]

Supplementary figures and figure legends

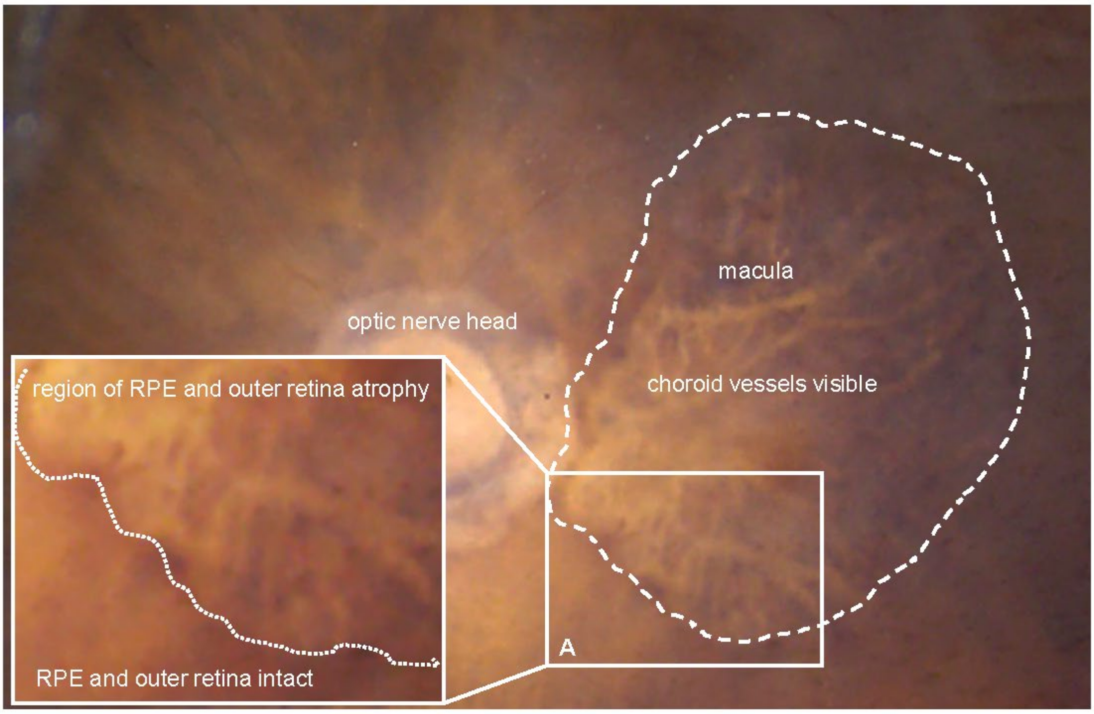

Supplementary Figure 1. Fundus image of post-mortem donor eye with geographic atrophy (GA) in the macula region.
The macula region (dashed line) shows evidence of RPE and outer retinal atrophy, with larger choroidal vessels clearly visible. Atrophy around the optic nerve head was also visible. The inset shows the distinct boundary (dotted line) between atrophic and intact retina; RPE is seen to the left, and the atrophic area to the right. Sections used for immunostaining were taken from region A.

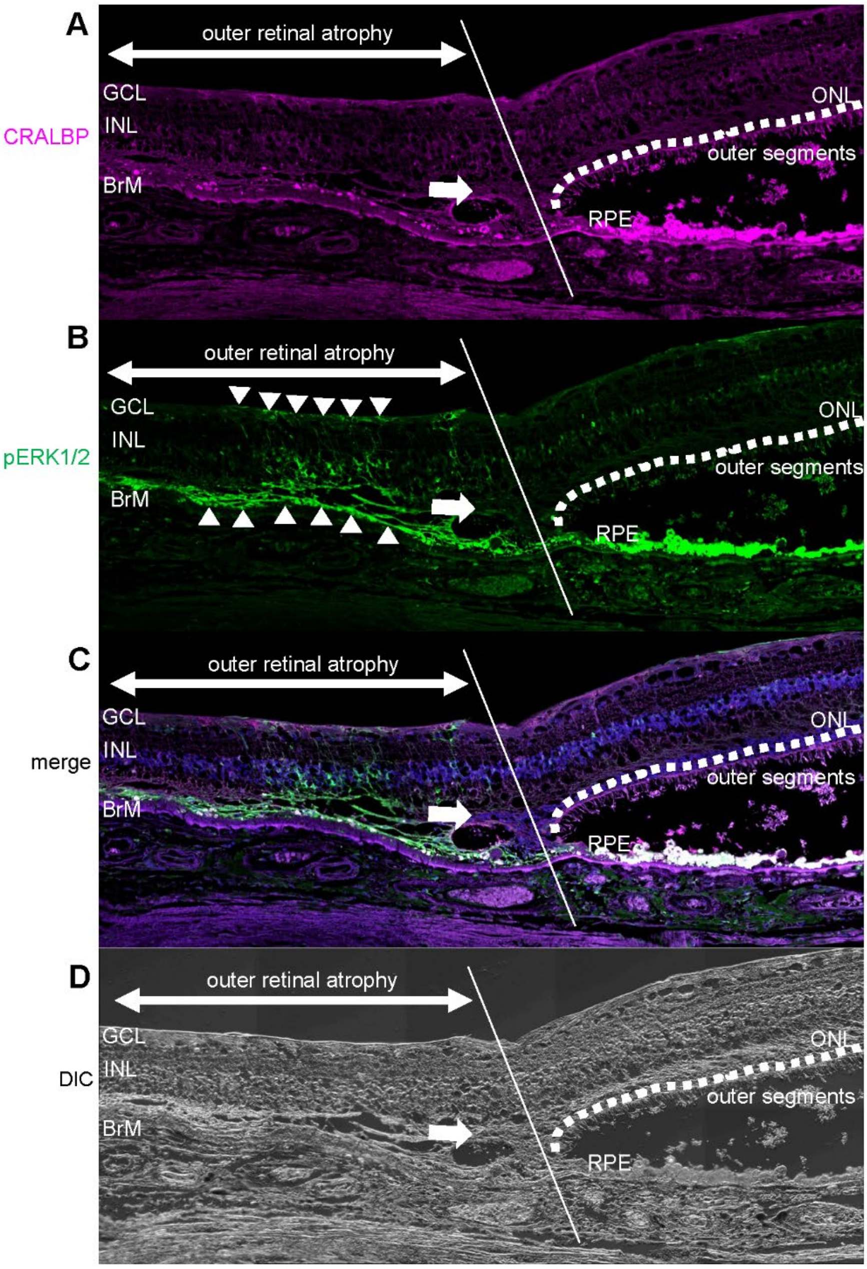

Supplementary Figure 2. MAPK(pErk1/2) activation in Müller cells in an eye with GA.
In this section, outer retinal atrophy is seen on the left side, with a loss of retinal photoreceptors, ONL and RPE. The white line indicates the edge of the atrophic lesion in the middle. The dotted white line on the right indicates the OLM extending to the right, where all retinal layers are visible without outer retinal atrophy. This is indicated on the merged and DIC images.
(A) Paraffin section of an eye with GA immunostained for CRALBP (magenta).
(B) Paraffin section of an eye with GA immunostained for pERK1/2 (green).
(C) Paraffin section of an eye with GA immunostained for CRALBP (magenta), pERK1/2 (green) and Hoechst (blue, cell nuclei staining) (merge). The ONL (photoreceptor nuclei) are

lost in the area of atrophy with BrM present in this region. To the right of this area, the OLM (dotted line) is visible with photoreceptor outer segments below and some detached outer segments can be seen, as well as the ONL visible. The RPE is seen on the right with some degeneration approaching the start of the edge of the atrophy (dotted line) and ONL above. White arrowheads indicate the area with outer retinal atrophy and activation of pERK1/2 (green).

(D) Paraffin section of an eye with GA imaged using DIC to show cell layers.

DIC: Differential Interference Contrast. GCL: ganglion cell layer. INL: inner nuclear layer. ONL: outer nuclear layer. OLM: outer limiting membrane. IS: inner segments. OS: outer segments. RPE: retinal pigment epithelium. CRALBP: cellular retinaldehyde-binding protein. GS: Glutamine Synthetase. GA: geographic atrophy. BrM: Bruch’s membrane. ERK1/2: extracellular signal-regulated kinase1/2. pEKR1/2: phosphorylated ERK1/2.

Scale Bar = 100 µm

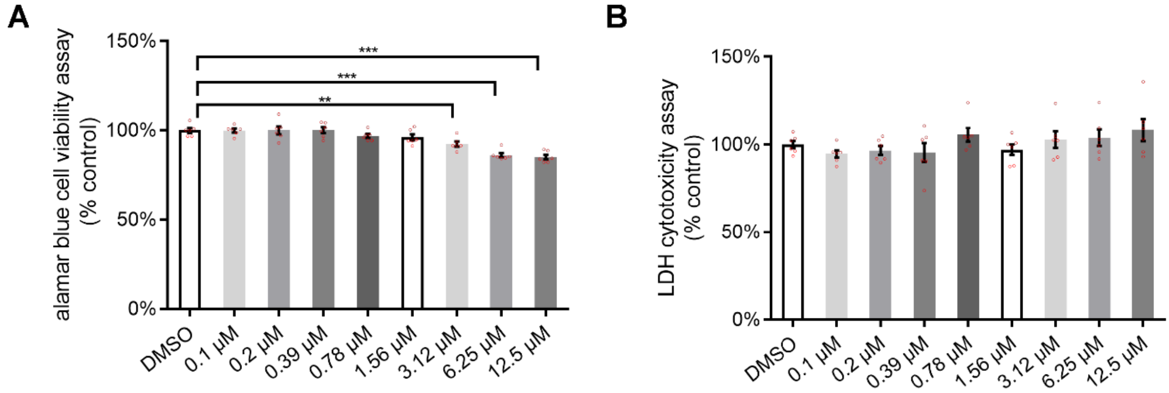

Supplementary Figure 3. Safety concentration and inhibition efficiency of PD98059 (pERK1/2 inhibitor) in Y79 cells

(A) Alamar Blue Assay of cell viability in photo-receptor like Y79 cells after 22 h treatment + 4h Alamar Blue Assay incubation with different concentrations of PD98059. Data are shown as the mean ± SEM. n = 6.

(B) LDH cell cytotoxicity assay in photo-receptor like Y79 cells after 22 h treatment with different concentrations of PD98059. Data are shown as the mean ± SEM. n = 6.

LDH: Lactate Dehydrogenase.

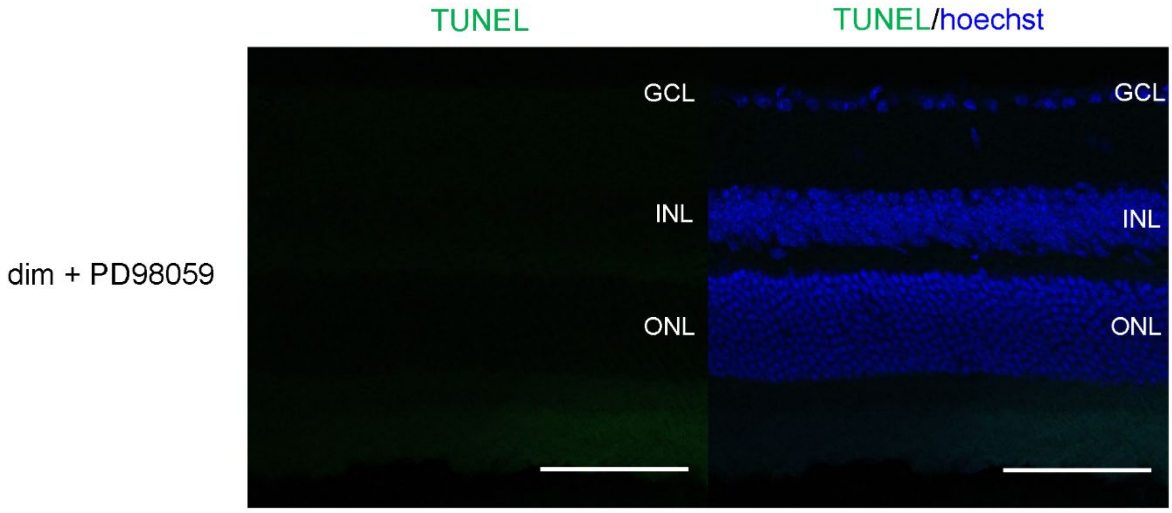

Supplementary Figure 4. Safety experiment for PD98059 (pERK1/2 inhibitor) in control mice.

Frozen sections of mice retinas stained for TUNEL (green) and Hoechst (blue, cell nuclei staining) in PD98059 injected control mice.

GCL: ganglion cell layer. INL: inner nuclear layer. ONL: outer nuclear layer. IS: inner segments. TUNEL: Terminal deoxynucleotidyl transferase dUTP nick end labelling.

Scale Bar = 100 μm

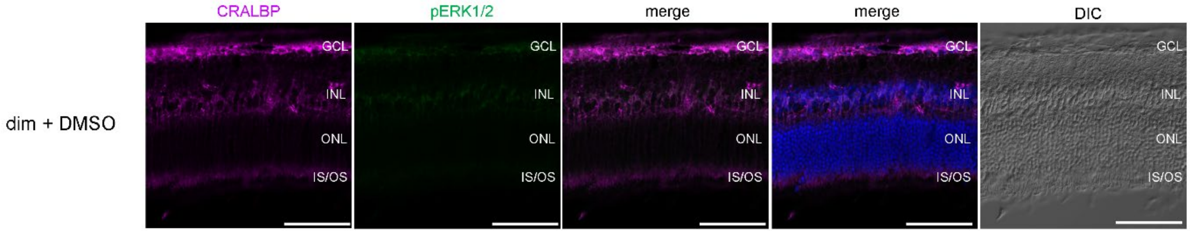

Supplementary Figure 5. CRALBP and pERK1/2 in DMSO injected mice eyes under dim light condition.

(A) Frozen sections of mice retinas stained for CRALBP(magenta), pERK1/2 (green) and Hoechst (blue, cell nuclei staining) in DMSO injected mice eyes under dim light condition. CRALBP: cellular retinaldehyde-binding protein. ERK1/2: Extracellular signal-regulated kinase 1/2. pEKR1/2: phosphorylated ERK1/2. DIC: Differential Interference Contrast. GCL: ganglion cell layer. INL: inner nuclear layer. ONL: outer nuclear layer. IS: inner segments. DMSO: dimethyl sulfoxide.

Scale Bar = 100 µm

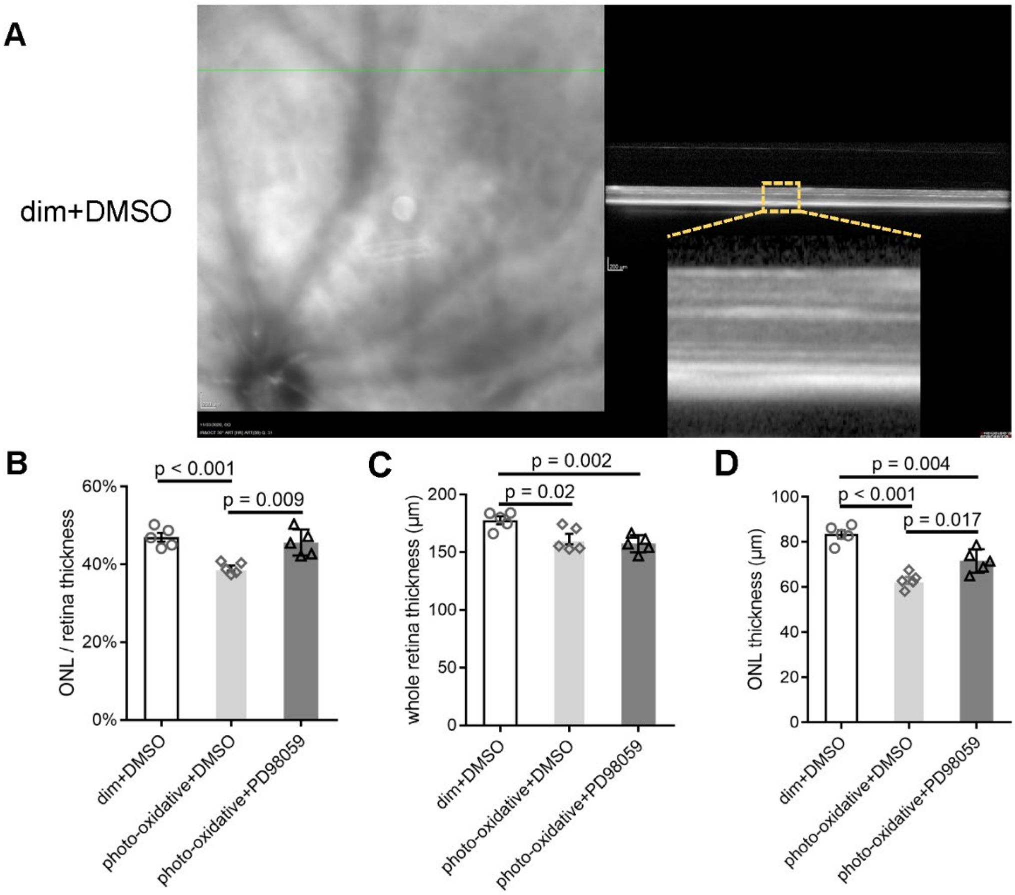

Supplementary Figure 6. OCT image of DMSO injected mice under dim light condition.

(A) Representative OCT images in DMSO injected mice under dim light condition and blow-ups.

(B-D) Quantification of (C) whole retina thickness, (D) ONL thickness and (B) their ratio in DMSO injected mice under dim light condition and DMSO or PD98059 injected photo-oxidative damage mice. Data are shown as the mean ± SEM. n = 5.

ONL: outer nuclear layer. DMSO: dimethyl sulfoxide.

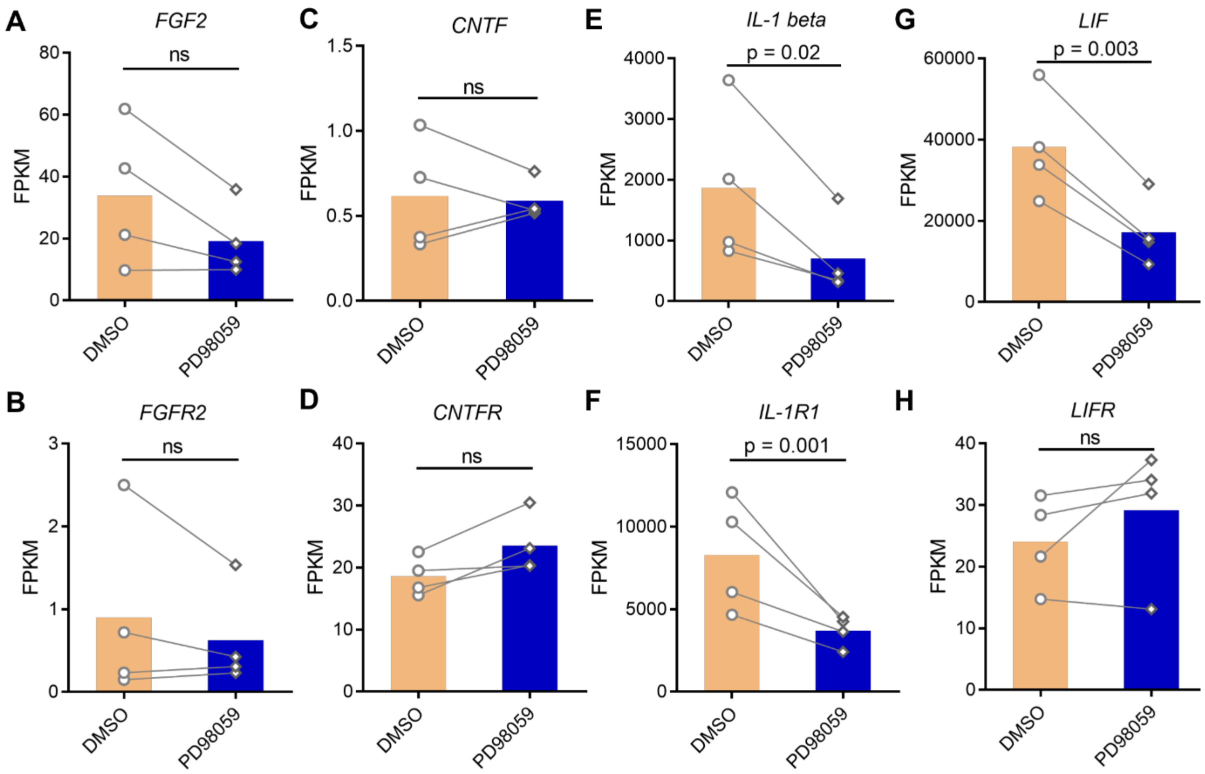

Supplementary Figure 7. RNA sequence quantification of neuroprotective factors in response to PD98059 (pERK1/2 inhibitor) in the 24 h cultured human retinal explant.

(A) RNA sequencing FPKM values for *FGF2* in 24 h cultured human retinal explant with DMSO or PD98059. n = 4.

(B) RNA sequencing FPKM values for *FGFR2* in 24 h cultured human retinal explant with DMSO or PD98059. n = 4.

(C) RNA sequencing FPKM values for *CNTF* in 24 h cultured human retinal explant with DMSO or PD98059. n = 4.

(D) RNA sequencing FPKM values for *CNTFR* in 24 h cultured human retinal explant with DMSO or PD98059. n = 4.

(E) RNA sequencing FPKM values for *IL-1 beta* in 24 h cultured human retinal explant with DMSO or PD98059. n = 4.

(F) RNA sequencing FPKM values for *IL-1R1* in 24 h cultured human retinal explant with DMSO or PD98059. n = 4.

(G) RNA sequencing FPKM values for *LIF* in 24 h cultured human retinal explant with DMSO or PD98059. n = 4.

(H) RNA sequencing FPKM values for *LIFR* in 24 h cultured human retinal explant with DMSO or PD98059. n = 4.

FPKM: Fragments Per Kilobase of transcript per Million mapped reads. *FGF: fibroblast growth factor. FGFR: fibroblast growth factor receptor. CNTF: ciliary neurotrophic factor. CNTFR: ciliary neurotrophic factor receptor. IL-1 beta: interleukin-1 beta. IL-1R1:interleukin 1 receptor type 1. LIF: leukemia inhibitory factor. LIFR: leukemia inhibitory factor receptor.* RNA: ribonucleic acid.
